# Multifunctional polyketide synthase genes identified by genomic survey of the symbiotic dinoflagellate, *Symbiodinium minutum*

**DOI:** 10.1186/s12864-015-2195-8

**Published:** 2015-11-14

**Authors:** Girish Beedessee, Kanako Hisata, Michael C. Roy, Noriyuki Satoh, Eiichi Shoguchi

**Affiliations:** Marine Genomics Unit, Okinawa Institute of Science and Technology Graduate University, Onna, Okinawa, 904-0495 Japan; Imaging and Instrumental Analysis Section, Okinawa Institute of Science and Technology Graduate University, Onna, Okinawa, 904-0495 Japan

**Keywords:** Gene diversification, Horizontal gene transfer, Spliced-leader trans-splicing, Polyketide synthase, Bacterial PKS, NRPS, Zooxanthellamide D, *Symbiodinium minutum*, Dinoflagellates, Genome-wide survey

## Abstract

**Background:**

Dinoflagellates are unicellular marine and freshwater eukaryotes. They possess large nuclear genomes (1.5–245 gigabases) and produce structurally unique and biologically active polyketide secondary metabolites. Although polyketide biosynthesis is well studied in terrestrial and freshwater organisms, only recently have dinoflagellate polyketides been investigated. Transcriptomic analyses have characterized dinoflagellate polyketide synthase genes having single domains. The Genus *Symbiodinium,* with a comparatively small genome, is a group of major coral symbionts, and the *S. minutum* nuclear genome has been decoded.

**Results:**

The present survey investigated the assembled *S. minutum* genome and identified 25 candidate polyketide synthase (PKS) genes that encode proteins with mono- and multifunctional domains. Predicted proteins retain functionally important amino acids in the catalytic ketosynthase (KS) domain. Molecular phylogenetic analyses of KS domains form a clade in which *S. minutum* domains cluster within the protist Type I PKS clade with those of other dinoflagellates and other eukaryotes. Single-domain PKS genes are likely expanded in dinoflagellate lineage. Two PKS genes of bacterial origin are found in the *S. minutum* genome. Interestingly, the largest enzyme is likely expressed as a hybrid non-ribosomal peptide synthetase-polyketide synthase (NRPS-PKS) assembly of 10,601 amino acids, containing NRPS and PKS modules and a thioesterase (TE) domain. We also found intron-rich genes with the minimal set of catalytic domains needed to produce polyketides. Ketosynthase (KS), acyltransferase (AT), and acyl carrier protein (ACP) along with other optional domains are present. Mapping of transcripts to the genome with the dinoflagellate-specific spliced leader sequence, supports expression of multifunctional PKS genes. Metabolite profiling of cultured *S. minutum* confirmed production of zooxanthellamide D, a polyhydroxy amide polyketide and other unknown polyketide secondary metabolites.

**Conclusion:**

This genomic survey demonstrates that *S. minutum* contains genes with the minimal set of catalytic domains needed to produce polyketides and provides evidence of the modular nature of Type I PKS, unlike monofunctional Type I PKS from other dinoflagellates. In addition, our study suggests that diversification of dinoflagellate PKS genes comprises dinoflagellate-specific PKS genes with single domains, multifunctional PKS genes with KS domains orthologous to those of other protists, and PKS genes of bacterial origin.

**Electronic supplementary material:**

The online version of this article (doi:10.1186/s12864-015-2195-8) contains supplementary material, which is available to authorized users.

## Background

Dinoflagellates are unicellular eukaryotes found in both marine and freshwater environments. Some are crucial symbionts of reef-building corals, and others sometimes cause toxic algal blooms [1]. Dinoflagellates are rich sources of structurally unique and bioactive secondary metabolites and are of interest to natural product chemists, biologists, and ecologists. These metabolites are unique in size, structure, and potency, and many are of polyketide origin [2–4].

Dinoflagellate toxins have been classified into three main categories: (i) polycyclic polyethers, (ii) macrolides, and (iii) linear polyethers [2, 4]. The majority of these compounds display remarkable biological activities, including ion channel modulation, phosphatase inhibition, hemolysis, mycotoxicity, and cytotoxicity [5–7]. One possible explanation for their high potency is to compensate for high dilution when they are released into the water [8]. Much is known regarding the biosynthesis of polyketides from terrestrial and freshwater organisms; however, only in the last decade have dinoflagellate polyketides been investigated.

Polyketides are synthesized by specific enzymes called polyketide synthases, through a series of condensation and reduction reactions involving at least three protein domains. These include ketosynthase (KS), acyl transferase (AT), and acyl carrier protein (ACP) (PP-binding) domains. In addition, polyketide synthesis may involve three optional domains: ketoreductase (KR), dehydratase (DH), and enoylreductase (ER) [9]. In 2008, full-length transcripts of Type I-like, modular PKS were sequenced from *Karenia brevis*, with seven out of eight transcripts containing single PKS domains, a feature typical of Type II PKS [10]. Eichholz et al. [11] characterized five transcripts for Type I-like, PKS-encoding KS proteins that are expressed as monofunctional units, from the dinoflagellates, *Alexandrium ostenfeldii* and *Heterocapsa triquetra*. Transcriptomic analysis of the non-toxic *Heterocapsa circularisquama*, revealed 61 polyketide synthase-encoding expressed sequence tags (EST) contigs, including one contig with two domains (KS-KR) [12]. Similar analysis revealed Type I-like polyketide synthases in the toxic dinoflagellate, *Gambierdiscus polynesiensis*, the main producer of ciguatoxins [13]. Meyer et al*.* [14] reported finding all genes essential for polyketide toxin synthesis in *Azadinium spinosum,* known to produce azaspiracid toxins. Recently, Kohli et al*.* catalogued 162 unique transcripts encoding complete KS domains in two species of *Gambierdiscus*, which are putatively involved in polyketide biosynthesis [15].

Among marine dinoflagellates, the Genus *Symbiodinium* includes major coral symbionts that are also associated with other invertebrate taxa (Porifera, Mollusca, and Platyhelminthes) [16, 17]. The draft genome of *S. minutum,* encoding ~42,000 protein-coding genes, has provided an opportunity for better understanding of its PKS system [18]. Snyder et al*.* [19] reported Type I PKSs in several dinoflagellates, including *Symbiodinium* sp.; however, there has been no detailed survey of genes involved in polyketide synthesis in *S. minutum*. We probed the *S. minutum* genome with respect to enzymes involved in polyketide synthesis and phylogenetically analyzed the KS domains of PKSs. We found a non-ribosomal peptide synthetase-polyketide synthase (NRPS-PKS) hybrid and confirmed that PKSs of *S. minutum* belong to the protistan Type I PKS group, along with some unexpected sequences associated with a bacterial clade.

## Results

### Diversification of KS domain-containing genes in the *S. minutum* genome

**Fig. 1:**
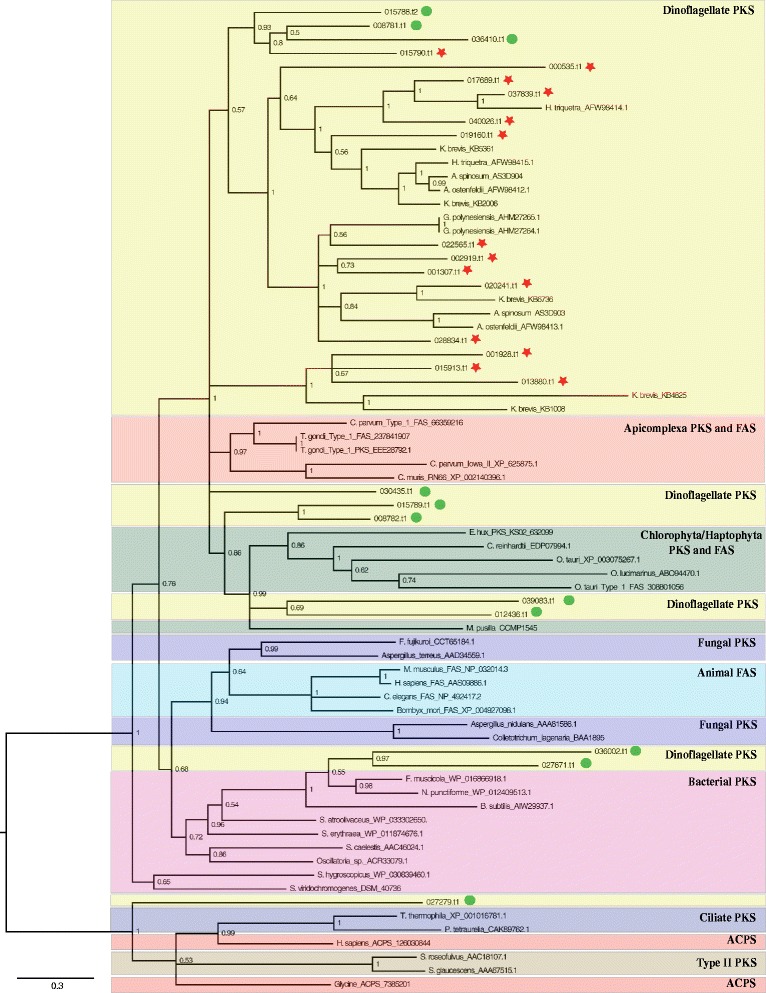
A molecular phylogenetic tree of Type I KS domains from prokaryotic and eukaryotic PKS and FAS, analyzed by Bayesian inference, reveals the diversification of the KS domain gene family. *Symbiodinium minutum* possesses genes belonging to three major groups within this gene family. Type II PKS and acyl carrier protein synthases (ACPS) were used as outgroups. Numbers at nodes indicate posterior probabilities. Details regarding *S. minutum* sequences are provided in Table 1. Red stars indicate *S. minutum* proteins with single PKS-related domains. Green circles indicate *S. minutum* proteins with multiple PKS-related domains. Dinoflagellate KSs (yellow) are classified as a well-supported group within the protistan Type I PKS clade.

In total, 65 genes with ketoacyl synthase domains (Pfam IDs: PF00109) were screened from the predicted 41,925 genes in the *S. minutum* genome (http://marinegenomics.oist.jp/genomes/gallery). Using BLASTP searches, we also checked *S. minutum* genes similar to reported PKSs and confirmed the aligned sequences manually. After removing sequences for partial domains, 25 genes that encoded full KS domains in *S. minutum* were selected for sequence characterization (Table 1; see Additional file 1: Table S1) and molecular phylogenetic analysis. Sequence comparisons with KS domains showed that the most similar genes are those reported from other dinoflagellates, although several genes were unexpectedly most similar to bacterial (*Bacillus*) genes. Eleven KS domain-containing genes likely encode multifunctional proteins with other domains related to PKS synthesis (AT, ACP, KR, DH, and ER) (Table 1). Careful examination of the *S. minutum* genome identified 25 intron-rich genes for KS sequences (Table 1) that are expressed under standard culture conditions (see Additional file 1: Figure S1). Only one KS gene (symbB1.v1.2.039083.1) is likely to be more highly expressed than genes [18] for RNA polymerase (data not shown). Quantitative expression analysis under different conditions will be useful for functional predictions. An interesting feature was the presence of tandemly aligned KS genes (symbB1.v1.2.015790.t1, symbB1.v1.2.015788.t2 and symbB1.v1.2.015789.t1) on scaffold 1186.1, in addition to two KS genes on scaffold 514.1 (Table 1). Since domain combinations are not conserved completely, duplication and/or splitting are hypothesized as the mechanisms for these expansions (see Fig. 1). This hypothesis is not unreasonable, considering a recent report of dinoflagellates possessing *por* (protochlorophyllide oxidoreductase) gene duplicates [20]. Gene duplications can have metabolic advantages and can eventually become fixed in a population.

**Table 1:** KS domain-containing genes in *Symbiodinium minutum*

Gene ID	Total AA	domain^a^	BLASTP best hit of KS domain in NCBI database (Accession #) Organism	E-value	Identity/ Similarities (% AA)	Scaffold # of *S.minutum* genome V.1.2	Assembled transcriptome ID
symbB1.v1.2.000535.t1	4838	KS	GAE32993.1 *Bacillus hemicellulosilyticus* JCM 9152	2.00E-47	45/60	31.1	symbB1.EST_k37c20_20326
symbB1.v1.2.001307.t1	797	KS	AIW63287.1 *Azadinium spinosum*	5.00E-128	35/51	57.1	symbB1.EST_k37c20_6869
symbB1.v1.2.001928.t1	1164	KS	ABQ85796.1 *Karenia brevis*	3.00E-174	35/50	55.1	symbB1.comp17616_c0_seq1
symbB1.v1.2.002919.t1	1105	KS	ABQ85802.1 *Karenia brevis*	4.00E-123	35/49	160.1	symbB1.EST_k37c20_17447
symbB1.v1.2.008781.t1	2107	DH-KR-PP-PP-KS-DH	KFG59574.1 *Toxoplasma gondii RUB*	0	34/49	514.1	symbB1.comp69166_c0_seq1
symbB1.v1.2.008782.t1	1848	KR-PP-KS-KR-PP-KS	WP_044601528.1 *Candidatus magnetoglobus multicellularis*	1.00E-161	34/54	514.1	symbB1.comp53648_c0_seq1
symbB1.v1.2.012436.t1	10601	See Fig. 2	CAD29793.1 *Planktothrix agardhii NIVA-CYA*	4.00E-60	39/56	860.1	symbB1.comp70898_c0_seq1
symbB1.v1.2.013880.t1	1071	KS	ABQ85796.1 *Karenia brevis*	2.00E-95	28/47	991.1	symbB1.comp24939_c0_seq1
symbB1.v1.2.015788.t2	920	KR-PP-KS-AT	CDJ53564.1 *Eimeria brunetti*	6.00E-171	40/56	1186.1	symbB1.comp40305_c0_seq1
symbB1.v1.2.015789.t1	1216	KR-PP-KS	CDI87737.1 *Eimeria praecox*	2.00E-153	37/53	1186.1	symbB1.EST_k37c20_822
symbB1.v1.2.015790.t1	427	KS	WP_035347688.1 *Bacillus hemicellulosilyticus*	5.00E-97	47/64	1186.1	symbB1.comp52059_c0_seq1
symbB1.v1.2.015913.t1	1671	KS	ABQ85796.1 *Karenia brevis*	0	37/55	1171.1	symbB1.comp20639_c0_seq1
symbB1.v1.2.017689.t1	2261	KS	AIW63289.1 *Azadinium spinosum*	3.00E-155	48/62	1368.1	symbB1.EST_k37c20_9081
symbB1.v1.2.019160.t1	1068	KS	AIW63288.1 *Azadinium spinosum*	0	37/55	1555.1	symbB1.EST_k37c20_8679
symbB1.v1.2.020241.t1	656	KS	ABQ85802.1 *Karenia brevis*	1.00E-156	44/59	1693.1	symbB1.EST_k37c20_3634
symbB1.v1.2.022565.t1	1547	KS	ABQ85802.1 *Karenia brevis*	7.00E-136	35/48	2011.1	symbB1.EST_k37c20_6838
symbB1.v1.2.027279.t1	615	KS	XP_005823341.1 *Guillardia theta*	0	72/82	2789.1	symbB1.comp12619_c0_seq1
symbB1.v1.2.027671.t1	957	AM-PP-KS-AT	KGC29420.1 *Burkholderia pseudomallei*	0	40/54	2857.1	symbB1.EST_k37c20_5813
symbB1.v1.2.028834.t1	2431	KS	AFW98413.1 *Alexandrium ostenfeldii*	1.00E-147	37/53	3094.1	symbB1.EST_k37c20_17396
symbB1.v1.2.030435.t1	481	KS-AT	XP_008886813.1 *Hammondia hammondi*	1.00E-107	42/59	3430.1	symbB1.comp58270_c0_seq1
symbB1.v1.2.036002.t1	2987	AM-PP-KS-KR-DH	AAR87760.2 *Bacillus cereus*	2.00E-110	31/46	4981.1	symbB1.EST_k37c20_11234
symbB1.v1.2.036410.t1	3519	AM-KS-KR-PP-KS-DH-KR-PP-KS	AFE09917.1 *Corallococcus coralloides* DSM 2259	0	33/48	5132.1	symbB1.comp56297_c0_seq6
symbB1.v1.2.037839.t1	604	KS	AIW63289.1 *Azadinium spinosum*	0	61/72	5703.1	symbB1.comp5164_c0_seq1
symbB1.v1.2.039083.t1	980	AT-KS-PP	XP_005785854.1 *Emiliania huxleyi* CCMP1516	5.00E-58	45/55	6338.1	symbB1.EST_k37c20_7312
symbB1.v1.2.040026.t1	582	KS	AFW98411.1 *Alexandrium ostenfeldii*	0	68/81	6945.1	symbB1.comp4031_c0_seq1

^a^ *AM* AMP-binding, *AT* Acyltransferase, *DH* Dehydratase, *ER* Enoylreductase, *KR* Ketoreductase, *KS* Ketosynthase, *PP* PP-binding

Bayesian inference and maximum likelihood analysis of the 25 KS sequences were carried out with acyl carrier protein synthase (ACPS) and Type II PKS sequences as outgroups to understand relationships of KS sequences from *S. minutum* compared with those of other dinoflagellates. After alignment and trimming, a sequence of 235 amino acids (aa) was used for analysis. Molecular phylogenetic analyses significantly placed most sequences with those of other dinoflagellate Type I PKSs (Fig. 1). Bayesian inference clearly demonstrated that 22 *S. minutum* proteins and other protistan proteins were included in that clade (Fig. 1). There was strong Bayesian support (posterior probability: 1.00) for a ‘protistan’ clade of Type I PKS sequences comprising of apicomplexan, dinoflagellate, chlorophyte, and haptophyte sequences (Fig. 1). These major groups formed two separate sub-groups within the protistan clade. One sub-group with all existing sequences from *A. ostenfeldii, A. spinosum, G. polynesiensis, and H. triquetra*, included 10 proteins of *S. minutum* with one PKS-related domain (KS). The second sub-group, with chlorophyte/haptophyte proteins, contained four *S. minutum* sequences with multiple PKS-related domains. The third sub-group, with bacterial proteins, contained two *S. minutum* proteins (symbB1.v1.2.027671 and symbB1.v1.2.036002). Interestingly, the sequences were more closely related to cyanobacterial KS sequences than to other eukaryote sequences. A similar pattern has been reported in *K. brevis*, in which some sequences grouped with cyanobacterial proteins [21]. Dinoflagellate proteins were not found in clades of animal fatty acid synthase (FAS) and fungal PKS. It is worth mentioning that NRPS-PKS hybrid (symbB1.v1.2.012436.t1) was in a clade with PKS and FAS of chlorophyta/haptophyta (*Emiliania huxleyi, Ostreococcus tauri, Chlamydomonas reinhardii, Ostreococcus lucimarinus and Micromonas pusilla*), which encode the largest PKS protein in *S. minutum*. Maximum likelihood analysis provided additional support for a “protistan clade” containing Type I PKS sequences (Additional file 1: Figure S2).

To explore the possibility of spliced leader trans-splicing from a large transcriptome to single-domain, protein-coding transcriptomes, we mapped transcriptome data from the TSS (transcription start site). The mapping of SL (spliced leader)-removed TSS (red lines in Figure S1) showed that each of three gene models (symbB1.v1.2.000535 on scaffold 31, symbB1.v1.2.020241 on scaffold 1693, symbB1.v1.2.028834 on scaffold 3094) predicts two transcripts by SL trans-splicing (red arrows in Figure S1). They are genes with single PKS-related domains (Table 1; Fig. 1). Therefore, proteins with multiple PKS-related domains are likely to be expressed in cultured *S. minutum*.

### Presence of a hybrid NRPS-PKS gene in *S. minutum*

**Fig. 2:**
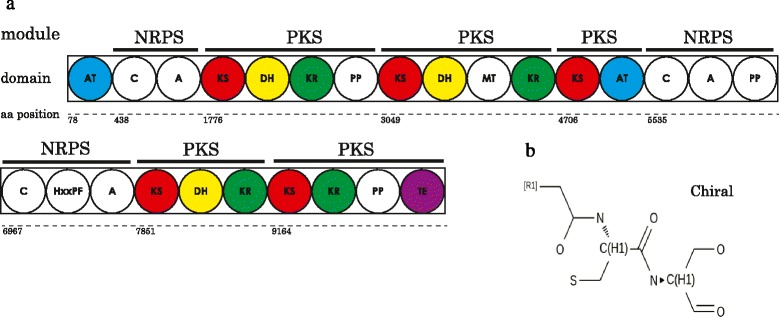
An unusual, very large NRPS-PKS protein is present in the *Symbiodinium minutum* genome. **a** Consensus schematic representation of the NRPS-PKS hybrid from *S. minutum* based on the PFAM database and anti-SMASH. Acyl carrier protein (ACP) is represented as phosphopantetheine (PP). The third A domain didn’t yield any prediction for a specific substrate using anti-SMASH. Amino acid (aa) positions show starting points for major domains. AT: acyl tansferase, C: condensation, A: adenylation, KS: ketosynthase, DH: dehydratase, KR: ketoreductase, MT: methyltransferase, TE: thioesterase **b** core scaffold of the hypothetical molecule, based on assumed PKS-NRPS co-linearity

One gene model of 10,601 amino acids was identified as a hybrid NRPS-PKS, based on PFAM domain analysis and confirmed by anti-SMASH (antibiotics & Secondary Metabolite Analysis Shell) [22]. It was composed of eight modules, three NRPSs, and five PKSs (Fig. 2a). The first NRPS is followed by three PKS modules, which contain at least a KS domain with different domain combinations. A second NRPS with condensation (C) and adenylation (A) is also present, followed by an additional NRPS-like assembly (C-HxxPF-A). Thioesterase (TE) domains are usually located in the final NRPS module where they catalyze product release [23]. Substrates of adenylation (A) domains in the NRPS module can be predicted based on residues in the binding pocket [24]. The reported A domains in NRPS proteins are responsible for recruiting amino acids into the final product. In this case, the first A domain has the sequence DLFNLSLI while the second A domain has DVWxFSLI. *In silico* methods were used to infer a hypothetical metabolite produced by the hybrid NRPS-PKS gene. Based on antiSMASH server prediction, the hybrid cluster could result in a natural product consisting of a core scaffold made from two amino acids (cysteine and serine) (Fig. 2b). I-TASSER [25] 3D protein structure prediction showed that the AT domain at the start of the cluster resembled an acyl-carrier-protein malonyltransferase (data not shown) and may provide malonyl groups for polyketide biosynthesis.

### Surveys of other PKS domains and conserved N-terminal sequences of *S. minutum* KS

BLAST screening of the *S. minutum* genome and comparisons of domain structure resulted in identification of candidates for all domains involved in polyketide synthesis: KS, AT, ACP (PP), KR, DH, and ER. First, we examined whether functionally important amino acid residues required for enzymatic activity (cysteine, histidine, and lysine) are present near the DTACSS-motif of *S. minutum* KS enzymes. We found that most sequences (21/25) contain these residues (see Additional file 2: Figure S3). Other domains can also be identified by comparing sequences and signature motifs: HxxxGxxxx with two active residues (histidine and proline) in DH [26], LxHxxxGGVG in ER with important residues (leucine, histidine, valine, and glycine) [26], GxGxxGxxxA in KR with three glycine and one alanine as essential residues [27] and the signature motif, GHSLG, in AT domains (see Additional file 2: Figure S4). The phylogenetic relationship of the PKS N-terminal region among dinoflagellates is shown in a maximum likelihood phylogeny (see Additional file 2: Figure S5). The tree reflects the same dinoflagellate resolution found in the KS-based phylogeny (Fig. 1) with one main sub-clade consisting of *A. spinosum*, *A. ostenfeldii*, *H. triqueta,* and *K. brevis*, one sub-clade comprising only one *S. minutum* and two *K. brevis* sequences, and a third clade consisting of three *S. minutum* sequences. Alignment of N-terminal regions revealed several conserved amino acid positions, including the highly conserved ExExGYLG in most dinoflagellates (see Additional file 2: Figure S5). In *Symbiodinium*, only one of the 25 sequences contained the signature GYLG and three other variants, DYLG, EYLG, and GYMG. Many variations were found in other sequences at the same position showing the diversified nature of the N-terminus in *S. minutum* (see Additional file 2: Figure S5).

### Identification of ZAD-D in cultured *S. minutum* by NanoLC-MS

ZAD (zooxanthellamide D) was identified based on high-resolution mass data (Table 2). A NanoLC-MS (positive ion) profile of the methanol extract of *S. minutum* showed ions at *m/z* 1072.60 (10.6 min) and 1050.57 for the [M + Na]^+^ and [M + H]^+^, respectively (Additional file 3: Figure S6). The ammonium adduct [M + NH_4_]^+^ at *m/z* 1067.59 (10.6 min) was also observed. It should be noted that other polyhydroxy molecules were also observed in the crude methanol extract, but none of them corresponded to other reported zooxanthella polyhydroxy molecules (Table 3).

**Table 2:** High-resolution MS of the target molecule, ZAD-D^45^

Ions	Obs. Mass (*m/z*)	Theo. Mass	Delta (mmu)	Formula
[M + H]^+^	1050.5657	1050.5632	2.46	C_54_H_84_O_19_N
[M + NH_4_]^+^	1067.5922	1067.5898	2.41	C_54_H_87_O_19_N_2_
[M + Na]^+^	1072.5476	1072.5452	2.46	C_54_H_83_O_19_NNa

**Table 3:** Compounds isolated from *Symbiodinium* sp. and their biological activities

Molecules	Reported m/z	Biological activity	References
Neosymbioimine	404.154	─	[54]
Norcarothenoids	630.3557	Growth-inhibitory activity against cancer cells	[55]
Symbiodinolide	2859	Activates N-type Ca ^2+^ channel	[56]
Symbioimine	378.1368	Anti-resorptive and Anti-inflammatory	[54]
Symbioramide	582	Ca^2+^-ATPase activator	[57]
Symbiospirols	1229	Inhibitory effect on protein kinase C	[58]
Zooxanthellatoxin A (ZT-A)	2872	Vasoconstrictive substance	[59]
Zooxanthellatoxin B (ZT-B)	2829.9	Vasoconstrictive substance	[60]
Zooxanthellamide A	2715.4	─	[61]
Zooxanthellamide B	2697.4	─	[62]
Zooxanthellamide C (ZAD-C)	2697.4016	Vasoconstrictive activity	[63]
Zooxanthellamide D (ZAD-D)	1072.6	Cytotoxicity against human carcinoma cell lines	[45], This study
Zooxanthellamine (ZA)	498.321	─	[64]
Zooxanthellactone (ZL)	327.2317	─	[65]

## Discussion

The KS domain is the most conserved domain of Type I PKS proteins and has divergent homologs, which permit comparative phylogenetic analysis of PKSs [28]. Our phylogenetic trees resolved previously reported clades [11, 14, 29]. Addition of novel sequences from *S. minutum* to the KS phylogenetic dataset provided evidence for three groups of dinoflagellate KS during PKS gene evolution. Other protist groups that diverged earlier also retain their KS evolutionary signatures and remain within well-supported clades, as shown by our analysis. A smaller clade comprising only *S. minutum* and *K. brevis* sequences showed alterations in their active sites (see Additional File 2: Figure S5). This topology may indicate a history of early gene duplication within the dinoflagellate clade.

As in PKSs, NRPSs also produce diverse secondary metabolites and have modular organizations with each module assuming specific functions. Formation of hybrid systems or clusters has been reported in bacteria [30, 31]. Fungal and bacterial NRPS and PKS have gained attention in recent years, mainly due to their complex evolutionary histories [32–34]. Lawrence et al*.* [35] provided evidence for horizontal gene transfer (HGT) of the hybrid NRPS-PKS gene from a putative bacterial donor in the *Burkholderiales* and suggested a HGT early in the history of the fungal Phylum Ascomycota. Bushley and Turgeon [34] identified *NPS* genes encoding NRPS and NRPS-like proteins in fungal genomes and suggested mechanisms for this modular architecture. In *Aspergillus* spp., genes involved in secondary metabolite biosynthesis tend to be located in subtelomeric regions, which may contribute to their rapid evolution [36, 37]. Gene transfer from cyanobacteria to dinoflagellates has been suggested by López-Legentil et al. [21] and this could explain the grouping of two *S. minutum* sequences with cyanobacterial sequences. The hybrid gene symbB1.v1.2.012436.t1 shares features with chlorophyte/haptophyte sequences used in our analysis. A data mining study found a surprisingly high number of hybrid NRPS-PKS gene clusters across three domains of life and this might be a consequence of long-term convergence between NRPS and PKS [38]. Our survey showed that the *S. minutum* genome encodes only one NRPS domain-containing gene. Hybrid NRPS-PKSs have been reported in other dinoflagellates (*K. brevis* [19] and *H. circularisquama* [12]). Dinoflagellate genomes are punctuated with a high number of simple and complex repeats and well known for frequent recombination events [39, 40]. Additionally, these genomes contain genes in high copy numbers, an indication of frequent gene duplication events during dinoflagellate evolution [41]. Shoguchi et al. [18] predicted that a total of 17,703 genes of *S. minutum* might have originated by gene duplication. It will be interesting to determine what types of natural products are synthesized by this NRPS-PKS gene and what role they do play in *S. minutum.*

Eichholz et al*.* [11] speculated that the N-terminus is related to the monofunctional nature of KS domains and may play a role in structural rearrangements, substrate docking, or protein-protein interactions. The N-termini of PKS multi-enzymes contain regularities in amino acid sequences. Recent studies have highlighted the potential role of these regions as “linkers” and their interactions with linker regions at the C-termini of PKS multi-enzymes [42, 43]. A low degree of conservation was noted within the N-terminal ExExGYLG signature sequence of *Symbiodinium* KS sequences. The GYLG conserved sequence has also been reported in *G. polynesiensis,* along with several variants (DYLG, HYLG, YYLG, GLLG and ALLG) [13]. Alteration of this signature has also been reported in *A. spinosum* (AFLG) [14]. Eichholz et al*.* [11] found that PKS domains are expressed as monofunctional units and that this feature may be unique to dinoflagellates. However, a transcript containing more than one domain has been reported in which one EST contig encoding two Type I PKS domains was found in the transcriptome of *H. circularisquama*, raising the possibility that there may be multi-modular PKS genes in dinoflagellates [12]. One feature suggesting this possibility is the presence of the ACP (PP arm), upstream of the KS domain. Such a group could serve as a swinging arm to present substrates to catalytic sites on PKS. Modular Type I PKS proteins have been reported in a closely related apicomplexan (*Cryptosporidium parvum*) and in a haptophyte (*Emiliana huxleyi*) with several different enzymatic domains arranged in distinct modules [29, 44]. Given that there is partial or complete absence of the conserved GYLG sequence in the N-terminus and that ACP precedes the KS domains, our genome-wide survey provides evidence for multifunctional PKS genes in the *Symbiodinium* genome along with monofunctional units.

ZAD-D is a linear polyhydroxylated polyketide and has been reported from *Symbiodinium* strain JCUCS-1 [45]. It is related to amphidinols isolated from the dinoflagellate, *Amphidinium* sp. [45]. This molecule is a polyhydroxy amide consisting of a C_22_-acid moiety and a C_32_-amine moiety; it furnishes three tetrahydropyran rings and six isolated butadiene chromophores. Apart from ZAD-D, other unknown polyhydroxy molecules were found in the methanol extract, and characterization of these unknown compounds could be interesting (see Additional File 3 Figure S6). Other natural products have been isolated from *Symbiodinium* sp. that displayed significant biological activities (Table 3). Hybrid NRPS-PKS systems are capable of incorporating both amino acids and short carboxylic acids into final products, eventually leading to greater chemical structural diversity. It is not yet known what type of natural products are synthesized by the hybrid NRPS-PKS reported here; further work is needed to characterize its end products as well as products of other PKSs in order to determine their role in *S. minutum*.

## Conclusions

We demonstrate that three structural types of enzymes for polyketide synthesis, single-domain PKS, multi-domain PKS, and NRPS-PKS hybrid, are present in the dinoflagellate, *S. minutum*. Based on the ketosynthase domain, dinoflagellate PKSs can be evolutionarily classified into three groups. It is not yet clear why *S. minutum* possesses a polyketide biosynthetic pathway and how these multifunctional PKS proteins have evolved. Genomic characterization of dinoflagellate PKS genes will likely provide insights for combinatorial biosynthesis of polyketides with wide range of applications. Due to large and complex dinoflagellate genomes, it is more difficult to perform comprehensive analyses; however, cultured *S. minutum*, which has a comparatively small genome, might provide further insights into these phenomena.

### Methods

#### Culture

*Symbiodinium minutum,* originally provided by Dr. Mary Alice Coffroth, of the University of New York, Buffalo, was cultured in autoclaved, artificial seawater containing 1X Guillard’s (F/2) marine-water enrichment solution (Sigma-Aldrich: G0154), plus three antibiotics, ampicillin (100 μg/mL), kanamycin (50 μg/mL), and streptomycin (50 μg/mL) [18]. Mass culturing was performed in a 2 L final volume by inoculating, and sampling at stationary phase one month later. A 12 h light/dark regime at 25 °C was maintained with a TOMY incubation chamber CLE-303.

### Transcriptome mapping

Transcriptome sequences of *S. minutum* were obtained in our previous study [18]. RNAseq reads have been deposited at DDBJ Sequence Read Archive (http://trace.ddbj.nig.ac.jp/dra/index_e.html) [DRR003865-DRR003871]. Reads from the Transcription start site (TSS) library have been also deposited at the DDBJ Sequence Read Archive [DRR023220-DRR023221]. Spliced leader (SL) sequences (DCCGTAGCCATTTTGGCTCAAG ) (D = T, A, or G) were removed from single TSS reads (99-bp). SL sequences were deleted to yield 77-bp reads that were then used for mapping onto the genome [18]. Reads were mapped onto *S. minutum* genome version 1 [DDBJ/EMBL/GenBank: DF239013-DF260911 (scaffolds)], using TopHat with default parameters [46]. SAMtools software was used for visualization of read coverage [47].

### Phylogenetic analysis

Amino acid sequences of KS domains were obtained from NCBI Genbank with additional sequences from Eichholz et al [11]. Type I and II PKS and FAS sequences, representing 39 different taxa, were used for Bayesian inference and maximum likelihood analysis. These represent major clades from prokaryote, fungal, animal, apicomplexan, haptophyte, and chlorophyte PKSs. Data included KS sequences from other dinoflagellates (*K. brevis*, *A. ostenfeldii, H. triqueta, G. polynesiensis,* and *A. spinosum*). The genome browser, MarinegenomicsDB (http://marinegenomics.oist.jp/genomes/gallery) [48] was accessed in order to retrieve PKS sequences. Multiple amino acid alignment was performed with the MUSCLE algorithm [49] included in MEGA 6 [50]. A maximum likelihood phylogenetic tree was generated with MEGA 6 using a Le-Gasquel amino acid replacement matrix with 1000 bootstraps. Bayesian inference was conducted with MrBayes v.3.2 [51] using the same replacement model and run for four million generations and four chains until the posterior probability approached 0.01. Statistics and trees were summarized using a burn-in of 25 % of the data [10]. Trees were edited using Figtree (http://tree.bio.ed.ac.uk/software/figtree/).

### Active sites of KS, AT, DH, ER, and KR domains and the N-terminus of the KS domain

In order to investigate whether active sites of major enzyme motifs were conserved, dinoflagellate sequences from NCBI were aligned with reference sequences from *A. ostenfeldii*, *A. spinosum*, *H. triquetra,* and *K. brevis*. For the N-termini of dinoflagellate KS sequences, other regions were separated from the KS domain and searched against the NCBI and PFAM databases [52]. Multiple alignments and phylogenies of the truncated sequences were calculated as described above for the KS domains.

### NRPS-PKS gene cluster

The antiSMASH server [22] was used to identify non-ribosomal peptide synthetase (NRPS) and polyketide synthase (PKS) domains. FASTA format protein sequences were used as input. Results were compared and further annotation was conducted using PFAM database [52]. NRPSpredictor2 was used to identify binding specificity of the A domains in the NRPS modules [53]. I-TASSER was used to identify the AT domain associated with the PKS module [25].

### Polyol extraction from *S. minutum* culture

Cultured cells were collected by centrifugation (9,000 g and 14,000 g, 10 min, 10 °C). After discarding the supernatant, the cell pellet was extracted with methanol (three times) at room temperature. Methanol (100 μL) was added to the biomass (37 mg, wet weight) followed by vortexing (1 min), sonication (10 min), and centrifugation (14,000 g, 10 min, 10 °C) to yield a methanol extract. The resulting clear solution was transferred to a new tube. By adding methanol (100 μL) to the residue, a second methanol extraction was carried out in the same fashion. The clear second methanol extract was combined with the first and stored at –30 °C. Additional methanol (100 μL) was added to the residue, vortexed (1 min), and kept overnight at room temperature. After centrifugation, the third methanol extract was pooled with the previous extracts (total 300 μL), and designated as the crude extract. To remove lipophilic materials, an aliquot (50 μL) of the crude extract was suspended in 50 μL water-methanol (90:10) containing 0.5 % formic acid. The suspension was vortexed (30 sec) and centrifuged (14,000 g, 10 min, 10 °C) to give a clean solution. The clean solution was transferred into a new tube (stock solution) and the insoluble part was discarded. The stock solution was kept at –30 °C before NanoLC-MS analysis or immediately analyzed after dilution.

### NanoLC-MS analysis of the *Symbiodinium* methanol extract

A Thermo Scientific hydride (LTQ Orbitrap) mass spectrometer was used for MS data collection. The mass spectrometer was equipped with an HPLC (Paradigm MS4, Michrom Bioresources Inc.), an auto-sampler (HTC PAL, CTC Analytics) and a nanoelectrospray ion source (NSI). High-resolution MS spectra were acquired at 60,000 resolution in FTMS mode (Orbitrap), full mass range *m/z* 400–2,000 Da with 200 °C capillary temperature, 1.9 kV spray voltage in positive ion mode. The lipid-depleted crude extract (stock solution) was diluted 1:50 by adding water-methanol (50:50) containing 0.25 % formic acid and separated on a capillary ODS column (50 × 0.18 mm, 3 μm, C_18_, Supelco). A 20-min gradient was used for polyol separation (10 % B for 0.0–2.0 min, 10–100 % B for 2.0–10.0 min, hold 100 % B for 10.0–15.0 min, equilibration 10 % B for 15.1–20.0 min; where solvent A is water:acetonitrile 98:2 and solvent B is water:acetonitrile 2:98, both containing 0.1 % formic acid; flow rate 2.0 μL/min, injection 2.0 μL loop).

### Availability of supporting data

The genome and transcriptome data of *Symbiodinium minutum* are available at http://marinegenomics.oist.jp/genomes/downloads?project_id=21.

## Additional files

Additional file 1:
**Table S1.** Predicted domains from transcriptome contigs **Figure S1.** Expression of KS domain-containing genes on scaffolds of *S. minutum*. Read coverages of RNAseq (gray line) on KS domain-containing genes (surrounded by green) show expression in our standard cultured conditions. In addition, the SL sequence containing reads (red line) from transcription start site (TSS) library suggest large multifunctional genes are expressed as a transcript that is not trans-spliced. Red arrows show trans-spliced sites, located internally in KS domain-containing genes. **Figure S2.** Molecular phylogenetic tree of Type I and Type II KS domains from prokaryotic and eukaryotic PKS and FAS, analyzed by maximum likelihood. Type II KS and acyl carrier protein synthases (ACPS) were used as outgroups. Bootstrap values ≥ 50 % are marked at appropriate nodes. Details regarding *S. minutum* sequences are provided in Table 1. (PDF 9386 kb) Additional file 2:
**Figure S3.** (A) Alignment of KS domains with those of other dinoflagellates and animal PKSs and FASs. Asterisks indicate conserved amino acids required for catalytic activity. (B) Additional *Symbiodinium* and *K. brevis* sequences that are consistent with previous reports. **Figure S4.** Alignments of motifs within AT, ACP, KR, ER, and DH domains. Active sites within the motifs are boxed with dashed lines. **Figure S5.** (A) Multiple alignment of the truncated, conserved, N-termini of dinoflagellate KSs (B) Additional *Symbiodinium* sequences with the divergent signatures. Lower panel: Maximum likelihood tree of the N-termini computed with 1000 bootstrap replicates. Bootstrap values ≥ 50 % are marked at appropriate nodes. (PDF 2705 kb) Additional file 3:
**Figure S6.** (A) NanoLC-MS (positive ion) profile of the methanol extract of *Symbiodinium minutum*. Top Chromatogram, Center Top: Extract ion (*m/z* 1072.60, 10.6 min), Center bottom: (*m/z* 1050.57, 10.6 min), Bottom: MS spectrum. (B) MS spectrum (positive ion) of the methanol extract (expanded). (PDF 145 kb)

## Abbreviations

ACP: Acyl carrier protein
ACPS: Acyl carrier protein synthase
antiSMASH: Antibiotics and Secondary Metabolite Analysis Shell
AT: Acyl transferase
DH: Dehydratase
ER: Enoylreductase
EST: Expressed sequence tags
FAS: Fatty acid synthase
HPLC: High-performance liquid chromatography
I-TASSER: Iterative Threading ASSEmbly Refinement
KR: Ketoreductase
KS: Ketosynthase
MT: Methyltransferase
NRPS-PKS: Non-ribosomal peptide synthetase-polyketide synthase
PKS: Polyketide synthase
TE: thioesterase
