# Supplementary material for: Multifunctional polyketide synthase genes identified by genomic survey of the symbiotic dinoflagellate, Symbiodinium minutum
Source: BMC Genomics. 2015 Nov 14;16:941. doi: 10.1186/s12864-015-2195-8 (PMC4647583; doi:10.1186/s12864-015-2195-8)

Table S1 Predicted domains from transcriptome contigs

Gene ID	Pfam domain	Assembled transcriptome ID	Pfam domain
symbB1.v1.2.000535.t1	ketoacyl-synt (PF00109), RVT_2 (PF07727), ketoacyl-synt (PF00109), Ketoacyl-synt_C (PF02801)	symbB1.EST_k37c20_20326	ketoacyl-synt (PF00109), Ketoacyl-synt_C (PF02801), PP-binding (PF00550), PP-binding (PF00550)
symbB1.v1.2.001307.t1	ketoacyl-synt (PF00109), Ketoacyl-synt_C (PF02801)	symbB1.EST_k37c20_6869	ketoacyl-synt (PF00109), Ketoacyl-synt_C (PF02801)
symbB1.v1.2.001928.t1	ketoacyl-synt (PF00109), Ketoacyl-synt_C (PF02801)	symbB1.comp17616_c0_seq1	ketoacyl-synt (PF00109), Ketoacyl-synt_C (PF02801)
symbB1.v1.2.002919.t1	ketoacyl-synt (PF00109), Ketoacyl-synt_C (PF02801)	symbB1.EST_k37c20_17447	ketoacyl-synt (PF00109), Ketoacyl-synt_C (PF02801)
symbB1.v1.2.008781.t1	Ketoacyl-synt_C (PF02801), PS-DH (PF14765), ADH_zinc_N (PF00107), KR (PF08659), PP-binding (PF00550), PP-binding (PF00550), ketoacyl-synt (PF00109), Ketoacyl-synt_C (PF02801), PS-DH (PF14765)	symbB1.comp69166_c0_seq1	PS-DH (PF14765), ADH_zinc_N (PF00107), KR (PF08659)
symbB1.v1.2.008782.t1	KR (PF08659), PP-binding (PF00550), ketoacyl-synt (PF00109), Ketoacyl-synt_C (PF02801), KR (PF08659), PP-binding (PF00550), ketoacyl-synt (PF00109), Ketoacyl-synt_C (PF02801)	symbB1.comp53648_c0_seq1	Ketoacyl-synt_C (PF02801), KR (PF08659)
symbB1.v1.2.012436.t1	Acyl_transf_1 (PF00698), Condensation (PF00668), AMP-binding (PF00501), ketoacyl-synt (PF00109), PS-DH (PF14765), KR (PF08659), PP-binding (PF00550), ketoacyl-synt (PF00109), Ketoacyl-synt_C (PF02801), PS-DH (PF14765), Methyltransf_23 (PF13489), KR (PF08659), ketoacyl-synt (PF00109), Ketoacyl-synt_C (PF02801), Acyl_transf_1 (PF00698), Condensation (PF00668), NRPS (PF08415), AMP-binding (PF00501), AMP-binding_C (PF13193), PP-binding (PF00550), Condensation (PF00668), HxxPF_rpt (PF13745), AMP-binding (PF00501), AMP-binding_C (PF13193), ketoacyl-synt (PF00109), Ketoacyl-synt_C (PF02801), PS-DH (PF14765), KR (PF08659), ketoacyl-synt (PF00109), Ketoacyl-synt_C (PF02801), KR (PF08659), PP-binding (PF00550), Thioesterase (PF00975)	symbB1.comp70898_c0_seq1	KR (PF08659), PP-binding (PF00550), Thioesterase (PF00975)
symbB1.v1.2.013880.t1	ketoacyl-synt (PF00109), Ketoacyl-synt_C (PF02801)	symbB1.comp24939_c0_seq1	ketoacyl-synt (PF00109), Ketoacyl-synt_C (PF02801)
symbB1.v1.2.015788.t2	KR (PF08659), PP-binding (PF00550), ketoacyl-synt (PF00109), Ketoacyl-synt_C (PF02801), Acyl_transf_1 (PF00698)	symbB1.comp40305_c0_seq1	PS-DH (PF14765), KR (PF08659), PP-binding (PF00550), ketoacyl-synt (PF00109), Ketoacyl-synt_C (PF02801), KR (PF08659), PP-binding (PF00550), ketoacyl-synt (PF00109), Ketoacyl-synt_C (PF02801), Acyl_transf_1 (PF00698)
symbB1.v1.2.015789.t1	KR (PF08659), PP-binding (PF00550), ketoacyl-synt (PF00109), Ketoacyl-synt_C (PF02801)	symbB1.EST_k37c20_822	PS-DH (PF14765), KR (PF08659), PP-binding (PF00550), ketoacyl-synt (PF00109), Ketoacyl-synt_C (PF02801), KR (PF08659), PP-binding (PF00550), ketoacyl-synt (PF00109)
symbB1.v1.2.015790.t1	ketoacyl-synt (PF00109), Ketoacyl-synt_C (PF02801)	symbB1.comp52059_c0_seq1	ADH_zinc_N (PF00107), KR (PF08659), PP-binding (PF00550), PP-binding (PF00550), ketoacyl-synt (PF00109), Ketoacyl-synt_C (PF02801)
symbB1.v1.2.015913.t1	ketoacyl-synt (PF00109), Ketoacyl-synt_C (PF02801)	symbB1.comp20639_c0_seq1	ketoacyl-synt (PF00109), Ketoacyl-synt_C (PF02801)
symbB1.v1.2.017689.t1	ketoacyl-synt (PF00109), Ketoacyl-synt_C (PF02801), Cupin_8 (PF13621)	symbB1.EST_k37c20_9081	ketoacyl-synt (PF00109), Ketoacyl-synt_C (PF02801)
symbB1.v1.2.019160.t1	ketoacyl-synt (PF00109), Ketoacyl-synt_C (PF02801)	symbB1.EST_k37c20_8679	ketoacyl-synt (PF00109), Ketoacyl-synt_C (PF02801)
symbB1.v1.2.020241.t1	ketoacyl-synt (PF00109), Ketoacyl-synt_C (PF02801)	symbB1.EST_k37c20_3634	ketoacyl-synt (PF00109), Ketoacyl-synt_C (PF02801)
symbB1.v1.2.022565.t1	ketoacyl-synt (PF00109), Ketoacyl-synt_C (PF02801), cNMP_binding (PF00027)	symbB1.EST_k37c20_6838	ketoacyl-synt (PF00109), Ketoacyl-synt_C (PF02801)
symbB1.v1.2.027279.t1	ketoacyl-synt (PF00109), Ketoacyl-synt_C (PF02801), Asp_Arg_Hydrox (PF05118)	symbB1.comp12619_c0_seq1	ketoacyl-synt (PF00109), Ketoacyl-synt_C (PF02801)
symbB1.v1.2.027671.t1	AMP-binding (PF00501), AMP-binding_C (PF13193), PP-binding (PF00550), ketoacyl-synt (PF00109), Ketoacyl-synt_C (PF02801), KAsynt_C_assoc (PF16197), Acyl_transf_1 (PF00698)	symbB1.EST_k37c20_5813	Thioesterase (PF00975), AMP-binding (PF00501), AMP-binding_C (PF13193), PP-binding (PF00550), ketoacyl-synt (PF00109), Ketoacyl-synt_C (PF02801), KAsynt_C_assoc (PF16197), Acyl_transf_1 (PF00698), Thioesterase (PF00975), KR (PF08659), PP-binding (PF00550)
symbB1.v1.2.028834.t1	Glyco_hydro_81 (PF03639), ketoacyl-synt (PF00109), Ketoacyl-synt_C (PF02801)	symbB1.EST_k37c20_17396	ketoacyl-synt (PF00109), Ketoacyl-synt_C (PF02801)
symbB1.v1.2.030435.t1	ketoacyl-synt (PF00109), Ketoacyl-synt_C (PF02801), Acyl_transf_1 (PF00698)	symbB1.comp58270_c0_seq1	ketoacyl-synt (PF00109), Ketoacyl-synt_C (PF02801), Acyl_transf_1 (PF00698)
symbB1.v1.2.036002.t1	AMP-binding (PF00501), ketoacyl-synt (PF00109), ketoacyl-synt (PF00109), KAsynt_C_assoc (PF16197), KR (PF08659), PS-DH (PF14765), HSP70 (PF00012)	symbB1.EST_k37c20_11234	AMP-binding (PF00501), ketoacyl-synt (PF00109), Ketoacyl-synt_C (PF02801), KAsynt_C_assoc (PF16197), KR (PF08659), PS-DH (PF14765), ADH_zinc_N (PF00107), PP-binding (PF00550), FSH1 (PF03959)
symbB1.v1.2.036410.t1	AMP-binding (PF00501), AMP-binding (PF00501), AMP-binding_C (PF13193), PP-binding (PF00550), ketoacyl-synt (PF00109), Ketoacyl-synt_C (PF02801), KR (PF08659), PP-binding (PF00550), ketoacyl-synt (PF00109), Ketoacyl-synt_C (PF02801), PS-DH (PF14765), KR (PF08659), PP-binding (PF00550), ketoacyl-synt (PF00109), Ketoacyl-synt_C (PF02801)	symbB1.comp56297_c0_seq6	PP-binding (PF00550), ketoacyl-synt (PF00109), Ketoacyl-synt_C (PF02801)
symbB1.v1.2.037839.t1	ketoacyl-synt (PF00109), Ketoacyl-synt_C (PF02801)	symbB1.comp5164_c0_seq1	ketoacyl-synt (PF00109), Ketoacyl-synt_C (PF02801)
symbB1.v1.2.039083.t1	Acyl_transf_1 (PF00698), ketoacyl-synt (PF00109), Ketoacyl-synt_C (PF02801), Ketoacyl-synt_C (PF02801), PP-binding (PF00550)	symbB1.EST_k37c20_7312	ketoacyl-synt (PF00109), Ketoacyl-synt_C (PF02801)
symbB1.v1.2.040026.t1	ketoacyl-synt (PF00109), Ketoacyl-synt_C (PF02801)	symbB1.comp4031_c0_seq1	ketoacyl-synt (PF00109), Ketoacyl-synt_C (PF02801)

Figure S1. Expression of KS domain-containing genes on scaffolds of *S. minutum*. Read coverages of RNAseq (gray line) on KS domain-containing genes (surrounded by green) show expression in our standard cultured conditions. In addition, the SL sequence containing reads (red line) from transcription start site (TSS) library suggest large multifunctional genes are expressed as a transcript that is not trans-spliced. Red arrows show trans-spliced sites, located internally in KS domain-containing genes.

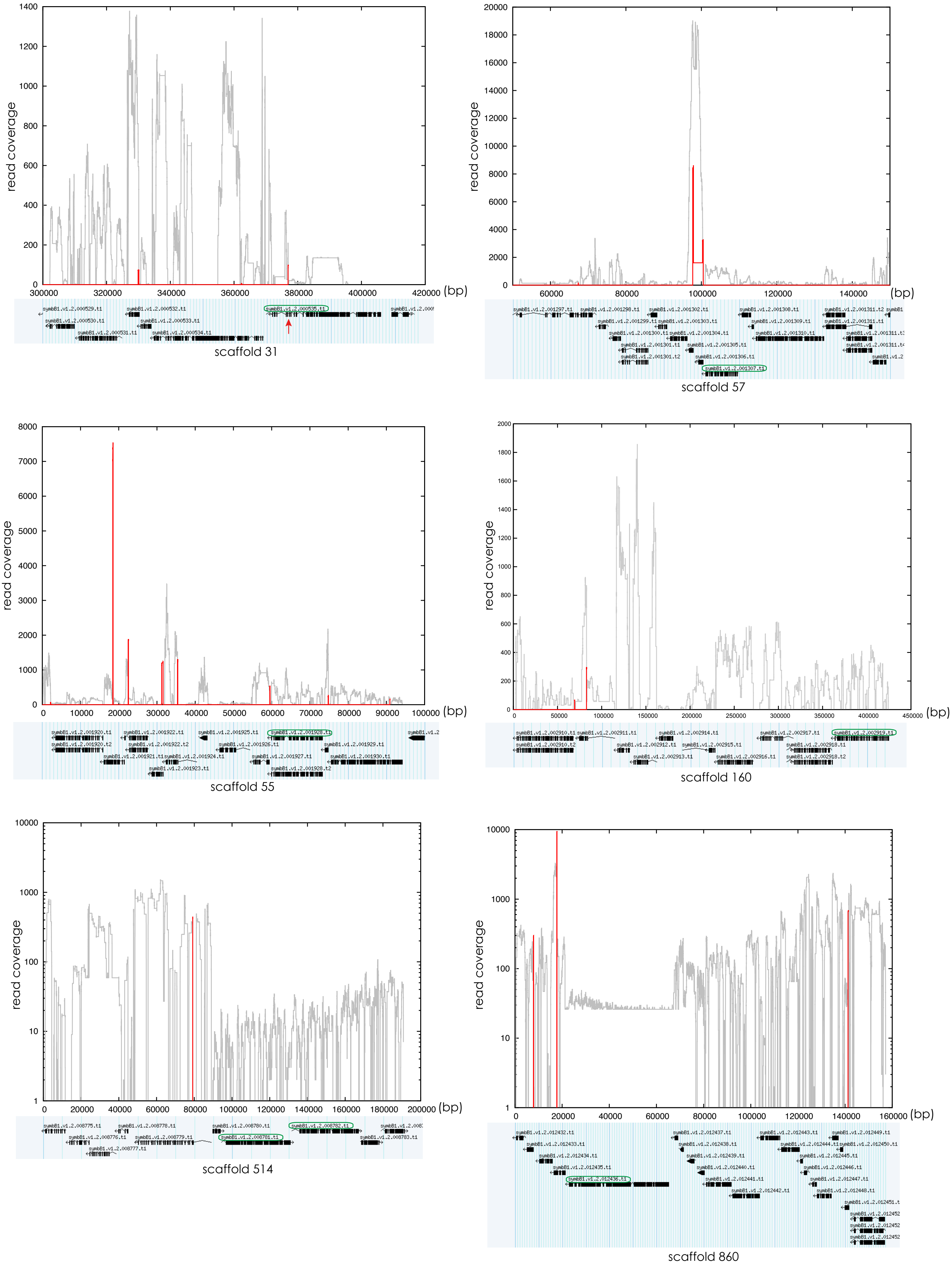

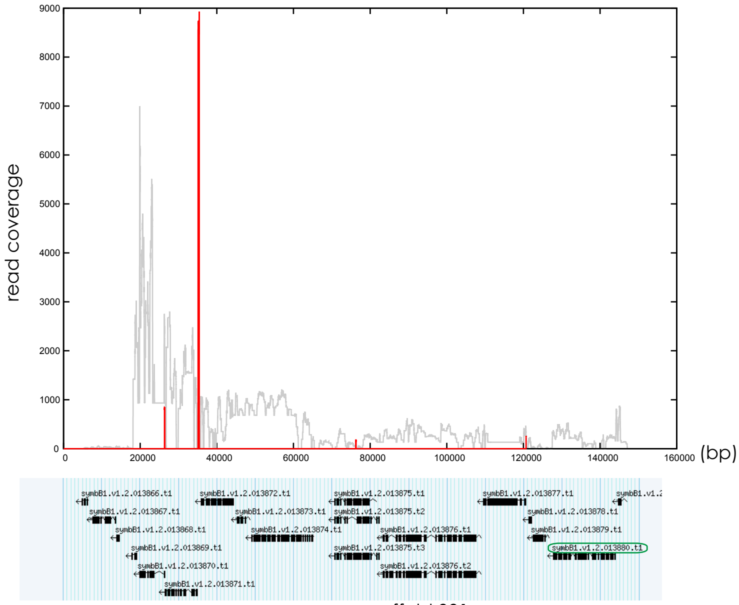

scaffold 991

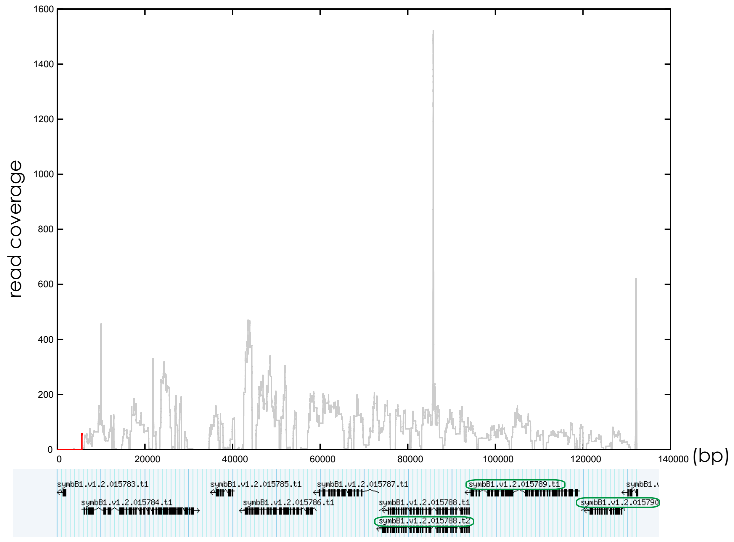

scaffold 1186

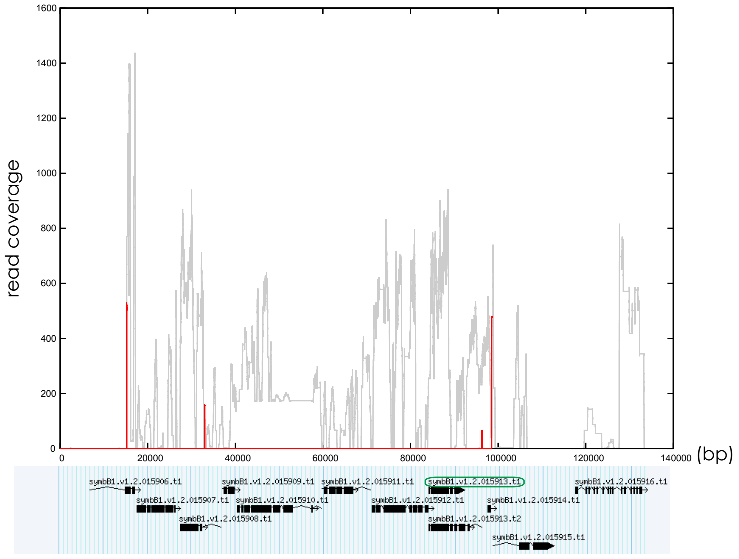

scaffold 1171

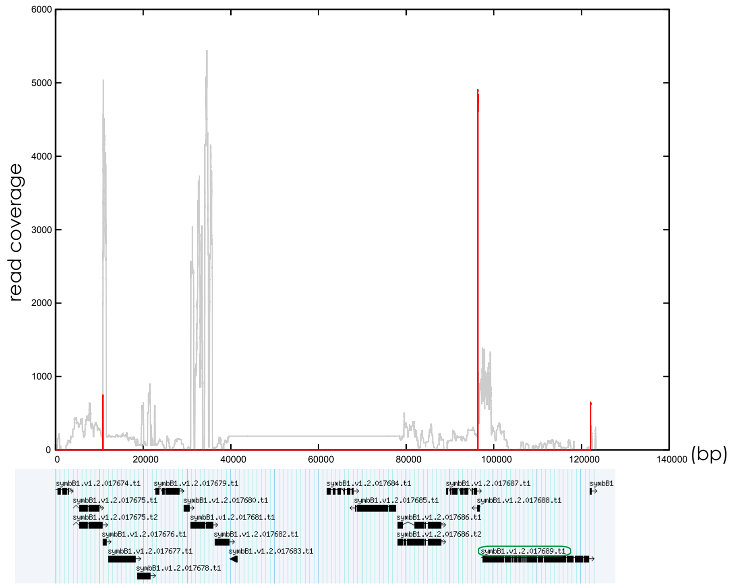

scaffold 1368

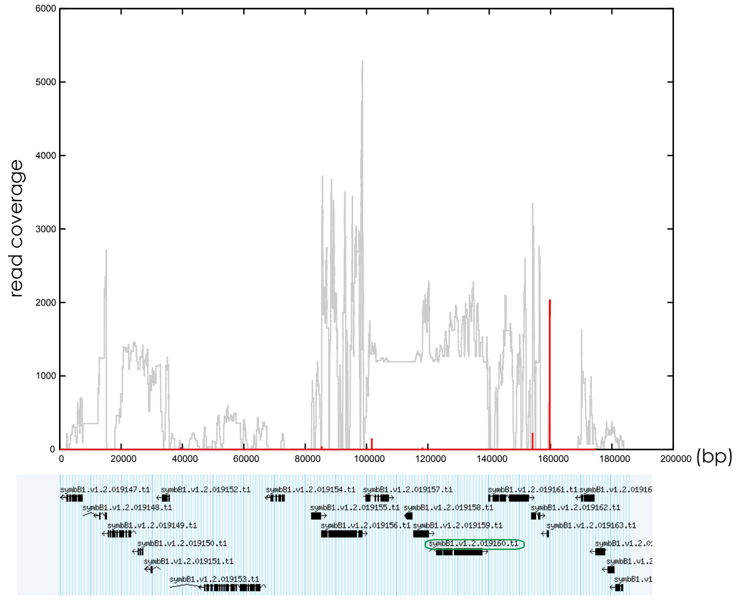

scaffold 1555

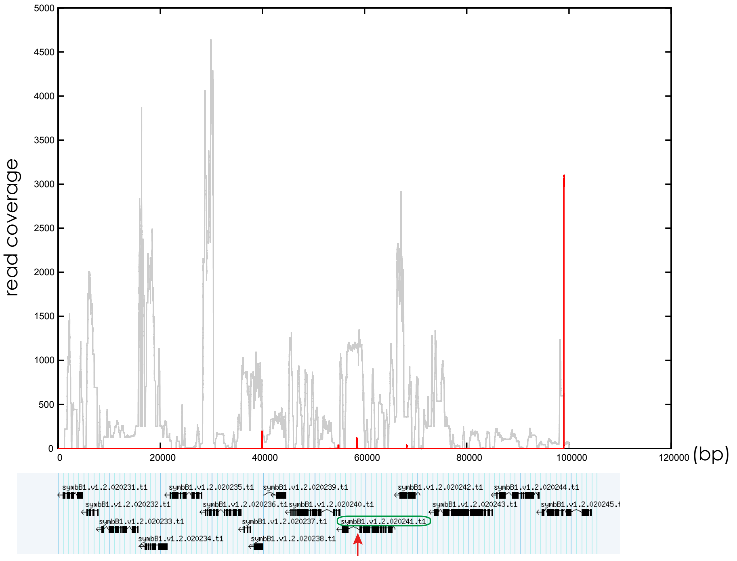

scaffold 1693

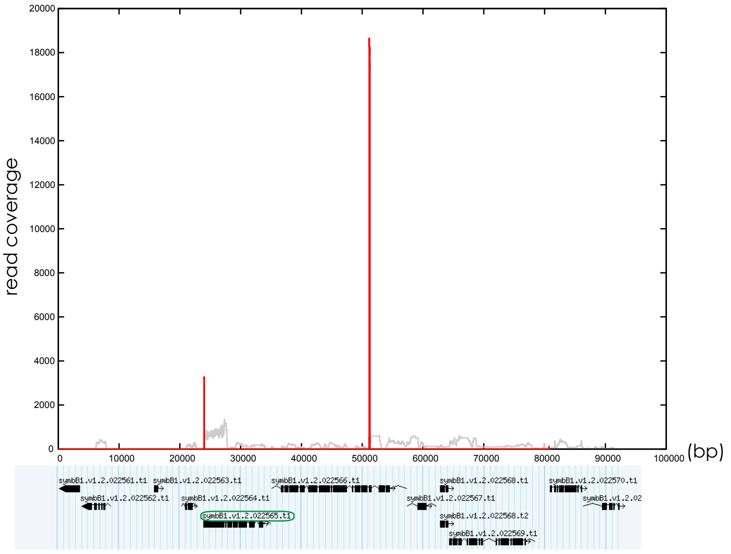

scaffold 2011

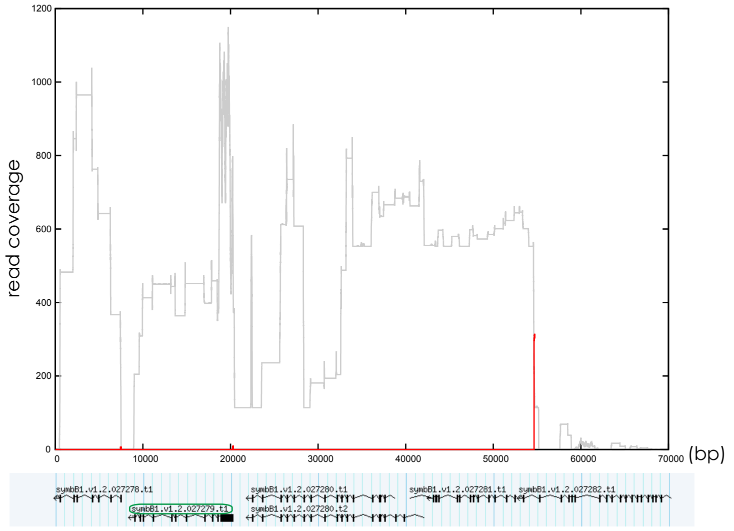

scaffold 2789

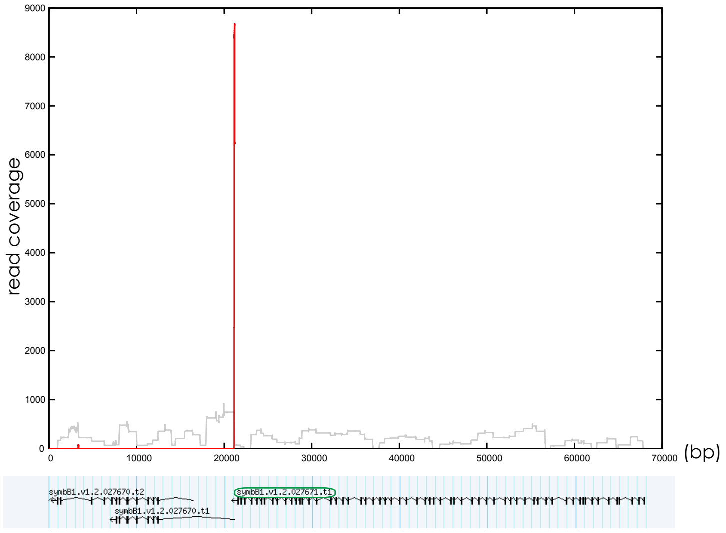

scaffold 2857

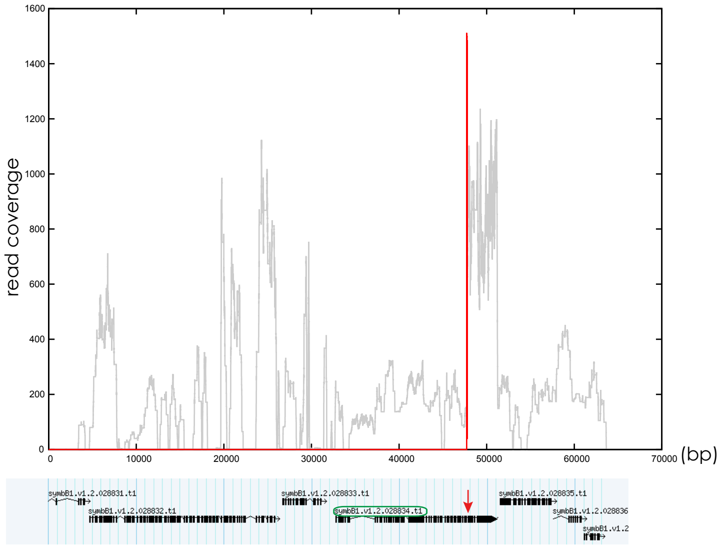

scaffold 3094

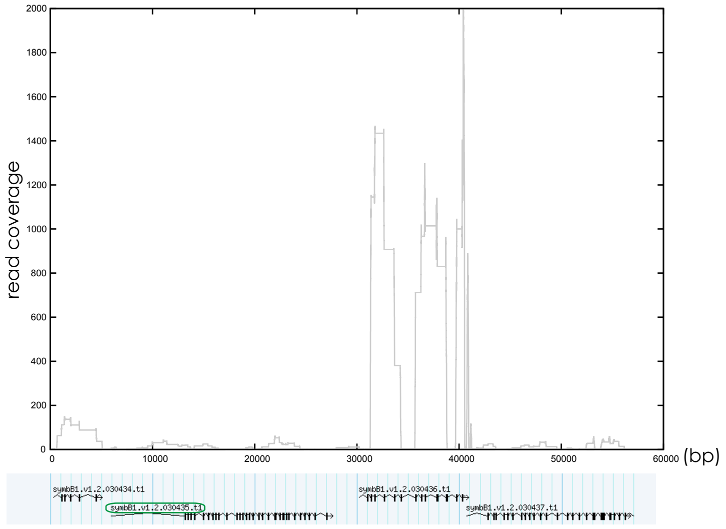

scaffold 3430

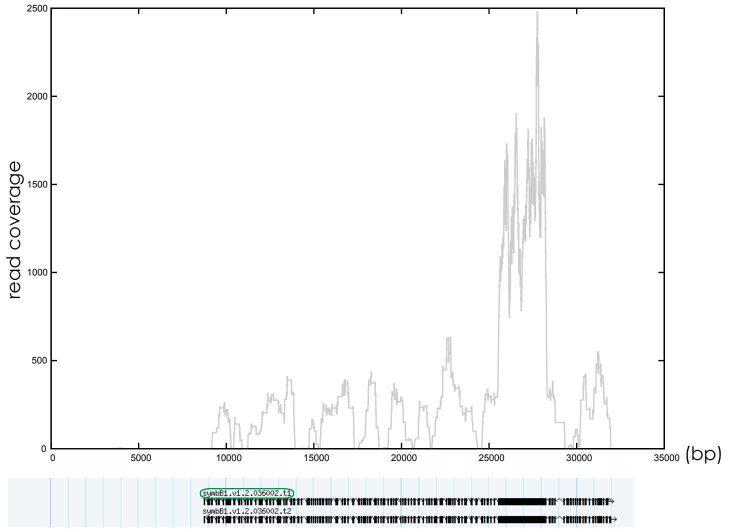

scaffold 4981

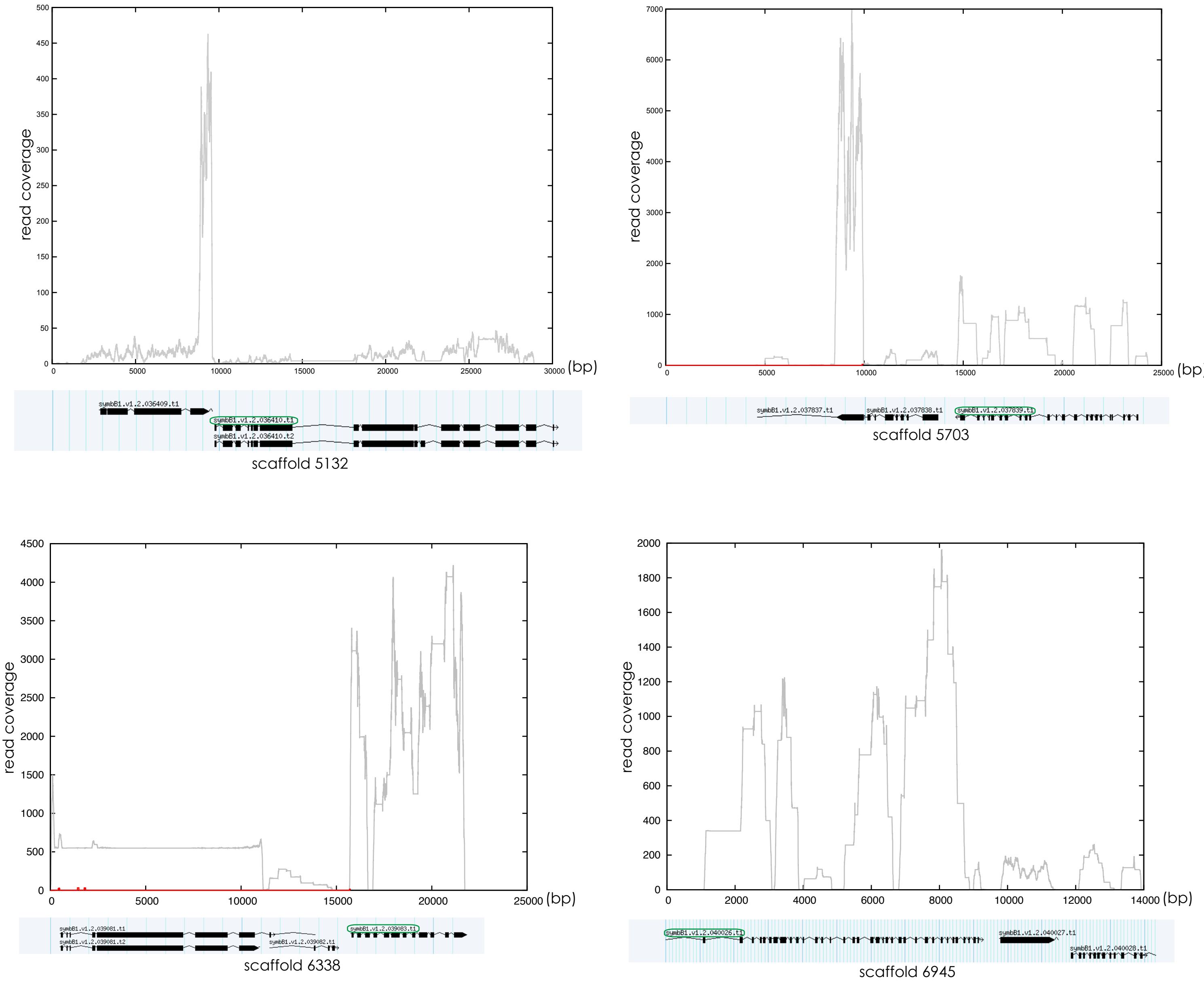

read coverage
(bp)
scaffold 5132
scaffold 5703
scaffold 6338
scaffold 6945

Figure S2. Molecular phylogenetic tree of Type I and Type II KS domains from prokaryotic and eukaryotic PKS and FAS, analyzed by a maximum likelihood. Type II KS and acyl carrier protein synthases (ACPS) were used as outgroups. Bootstrap values ≥ 50% are marked at appropriate nodes. Details regarding *S. minutum* sequences are provided in Table 1.

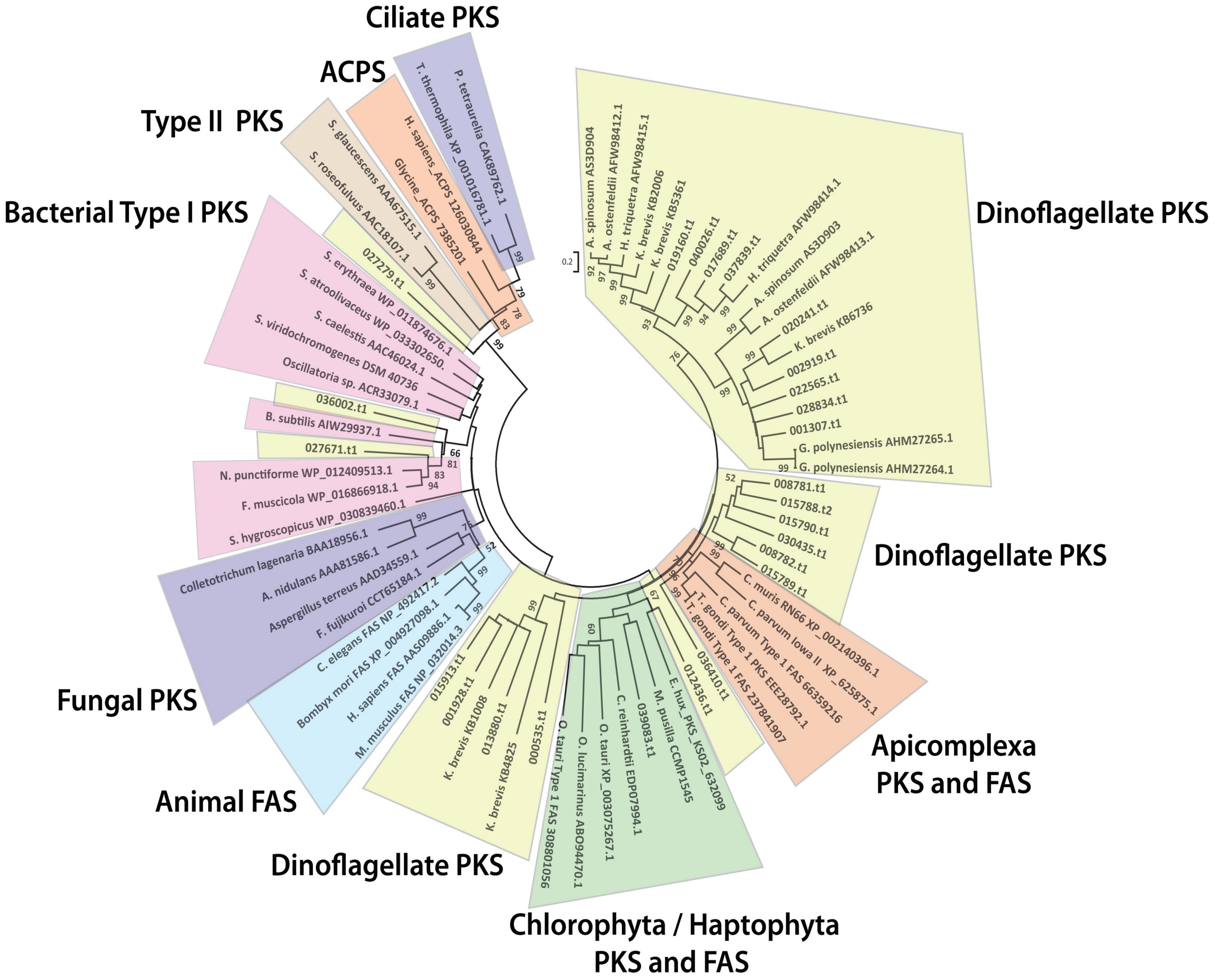

Supplement: Additional file 1: — Table S1. Predicted domains from transcriptome contigs Figure S1. Expression of KS domain-containing genes on scaffolds of S. minutum. Read coverages of RNAseq (gray line) on KS domain-containing genes (surrounded by green) show expression in our standard cultured conditions. In addition, the SL sequence containing reads (red line) from transcription start site (TSS) library suggest large multifunctional genes are expressed as a transcript that is not trans-spliced. Red arrows show trans-spliced sites, located internally in KS domain-containing genes. Figure S2. Molecular phylogenetic tree of Type I and Type II KS domains from prokaryotic and eukaryotic PKS and FAS, analyzed by maximum likelihood. Type II KS and acyl carrier protein synthases (ACPS) were used as outgroups. Bootstrap values ≥ 50 % are marked at appropriate nodes. Details regarding S. minutum sequences are provided in Table 1. (PDF 9386 kb) [file 12864_2015_2195_MOESM1_ESM.pdf]
