# Supplementary material for: Multifunctional polyketide synthase genes identified by genomic survey of the symbiotic dinoflagellate, Symbiodinium minutum
Source: BMC Genomics. 2015 Nov 14;16:941. doi: 10.1186/s12864-015-2195-8 (PMC4647583; doi:10.1186/s12864-015-2195-8)

Figure S3. (A) Alignment of KS domains with those of other dinoflagellates and animal PKSs and FASs. Asterisks indicate conserved amino acids required for catalytic activity. (B) Additional *Symbiodinium* and *K. brevis* sequences that are consistent with previous reports.

A

KS

					*						*										*							
**Bacillus subtilis polyketide synthase gi|703574048**	I	D	T	A	C	S	S	S	L	V	H	G	T	G	T	E	L	G	D	P	K	T	N	I	G	H	L	S
**Fusarium fujikuroi polyketide synthase gi|517312935**	I	D	T	A	C	S	S	T	L	T	H	G	T	G	T	K	V	G	D	P	K	S	N	I	G	H	L	E
**Homo sapiens fatty acid synthase gi|41584442**	L	D	T	A	C	S	S	S	L	M	H	G	T	G	T	K	V	G	D	P	K	S	N	M	G	H	P	E
**Bombyx mori fatty acid synthase-like gi|512910155**	V	D	T	A	C	S	S	S	M	F	H	G	T	G	T	K	V	G	D	P	K	S	N	M	G	H	S	E
**O. tauri polyketide synthase (ISS) gi|308800972**	I	D	T	A	C	S	S	G	L	C	H	G	T	G	T	A	L	G	D	P	K	T	S	C	A	H	S	E
**O. lucimarinus polyketide synthase gi|144576402**	V	D	T	A	C	S	S	S	L	V	H	G	T	G	T	S	L	G	D	P	K	P	R	A	G	H	C	E
**E.huxleyi CCMP1516 polyketide synthase gi|551577402**	Y	D	T	A	C	S	S	A	L	V	H	G	T	G	T	A	L	G	D	P	K	A	C	A	G	H	A	E
**T.gondii polyketide synthase TGGT1_411570**	I	D	S	A	C	S	S	S	L	V	H	G	T	G	T	S	L	G	D	P	K	S	R	I	G	H	T	E
**C.parvum Iowa II polyketide synthase gi|66357394**	I	D	T	A	C	S	S	S	L	V	H	G	T	G	T	P	L	G	D	P	K	P	N	I	G	H	L	E
**A.spinosum polyketide synthase gi|712037593**	I	D	T	A	C	S	S	S	L	V	H	G	T	G	T	S	L	G	D	P	K	S	N	V	A	H	S	E
**A.spinosum polyketide synthase gi|712037591**	A	D	T	A	C	S	A	S	L	V	H	G	T	G	T	A	L	G	D	P	K	A	H	V	G	H	E	E
**A.ostenfeldii Type I polyketide synthase gi|414091051**	V	D	T	A	C	S	A	S	L	V	H	G	T	G	T	A	L	G	D	P	K	S	H	V	G	H	E	E
**A.ostenfeldii Type I polyketide synthase gi|414091049**	I	D	T	A	C	S	S	S	L	V	H	G	T	G	T	S	L	G	D	P	K	S	N	V	A	H	G	E
**H.triquetra type I polyketide synthase gi|414091055**	I	D	T	A	C	S	S	S	L	V	H	G	T	G	T	S	L	G	G	P	K	S	N	M	A	H	G	E
**H.triquetra type I polyketide synthase gi|414091053**	A	D	T	A	C	S	A	S	L	A	H	G	T	G	T	S	L	G	D	P	K	T	T	T	G	H	L	E
**K.brevis KB2006 Type I polyketide synthase-like protein**	I	D	T	A	C	S	S	G	L	V	H	G	T	G	T	S	L	G	D	P	K	S	N	I	C	H	G	E
**K.brevis KB5361 Type I polyketide synthase-like protein**	I	D	T	A	C	S	A	S	L	V	H	G	T	G	T	S	L	G	D	P	K	S	N	I	G	H	C	E
**K.brevis KB6736 Type I polyketide synthase-like protein**	M	D	T	A	C	S	A	S	L	S	H	G	T	G	T	A	L	G	D	P	K	S	N	L	A	H	T	E
**symbB1.v1.2.040026.t1**	C	D	T	A	C	S	S	S	L	V	H	G	T	G	T	S	L	G	D	P	K	S	N	L	G	H	L	E
**symbB1.v1.2.039083.t1**	I	N	T	A	C	S	S	A	L	V	H	G	T	G	T	P	L	G	D	P	K	M	H	V	G	H	L	E
**symbB1.v1.2.037839.t1**	A	D	T	A	C	S	A	S	L	T	H	G	T	G	T	S	L	G	D	P	K	S	Q	T	G	H	L	E
**symbB1.v1.2.036410.t1**	I	D	A	A	C	S	S	S	M	V	H	G	T	G	T	K	L	G	D	P	K	S	N	V	G	H	L	E
**symbB1.v1.2.036002.t1**	V	Q	T	A	C	S	S	A	L	V	H	G	T	G	T	K	L	G	D	P	K	G	N	V	G	H	L	N
**symbB1.v1.2.030435.t1**	I	D	T	A	C	S	S	S	L	M	H	G	T	G	T	A	L	G	D	P	K	S	F	L	G	H	L	E
**symbB1.v1.2.028834.t1**	Y	D	T	A	C	S	A	S	L	V	H	G	T	G	T	A	L	G	D	P	K	S	N	I	A	H	L	E
**symbB1.v1.2.027671.t1**	V	N	S	A	C	S	S	A	L	V	H	C	T	G	T	R	I	G	D	G	K	G	N	I	A	H	A	N

symbB1.v1.2.027279.t1	I	S	S	A	C	S	T	G	A	H	H	G	T	S	T	P	I	G	D	V	K	S	M	S	G	H	A	L
symbB1.v1.2.022565.t1	I	D	T	A	C	S	A	S	L	V	H	G	T	G	T	A	L	G	D	P	K	S	N	I	G	H	T	E
symbB1.v1.2.020241.t1	T	E	T	A	C	S	S	S	L	S	H	G	T	G	T	A	L	G	D	P	K	S	N	L	G	H	T	E
symbB1.v1.2.019160.t1	V	N	T	A	C	S	A	S	L	V	H	G	T	G	T	S	L	G	D	P	K	P	N	L	G	H	L	E
symbB1.v1.2.017689.t1	C	D	T	A	C	S	A	S	L	T	H	G	T	G	T	S	L	G	D	P	K	S	N	I	G	H	L	E
symbB1.v1.2.015790.t1	V	D	T	A	C	S	S	A	L	V	H	G	T	G	T	P	L	G	D	P	K	S	N	C	G	H	L	E
symbB1.v1.2.015789.t1	V	D	T	A	C	S	S	T	I	V	H	G	T	G	T	A	L	G	D	P	K	S	N	I	G	H	L	E
symbB1.v1.2.015788.t2	M	D	T	A	C	S	S	S	L	V	H	G	T	G	T	A	L	G	D	P	K	T	N	V	G	H	L	E
symbB1.v1.2.008782.t1	I	D	T	A	C	S	S	T	L	V	H	G	T	G	T	A	L	G	D	P	K	S	V	I	A	H	L	E
symbB1.v1.2.008781.t1	C	D	T	A	C	S	S	S	L	V	H	G	T	G	T	A	L	G	D	P	K	T	N	I	A	H	L	E
symbB1.v1.2.002919.t1	L	D	T	A	C	S	S	A	L	V	H	G	T	G	T	A	L	G	D	P	K	S	H	I	G	H	L	E
symbB1.v1.2.001307.t1	A	E	T	A	C	S	S	S	L	V	H	G	T	G	T	A	L	G	D	P	K	T	N	L	G	H	L	E
symbB1.v1.2.012436.t1	V	D	T	A	C	S	S	S	L	I	H	A	T	G	T	W	F	G	D	Q	K	S	V	L	G	H	H	E

B

K.brevis KB6380 Type I polyketide synthase-like protein	Y	E	A	D	A	S	S	A	M	V	F	G	D	G	R	F	L	H	E	A	K	G	S	V	G	D	T	L
K.brevis KB4825 Type I polyketide synthase-like protein	A	A	T	G	A	A	P	S	V	V	S	G	T	G	H	F	F	C	D	A	K	S	N	V	G	N	S	I
symbB1.v1.2.015913.t1	I	D	T	D	A	S	S	G	A	T	Y	G	A	G	A	F	L	P	D	A	K	S	S	I	G	N	Q	I
symbB1.v1.2.013880.t1	I	D	C	D	L	S	S	S	A	M	H	A	E	G	A	K	L	S	D	S	K	T	N	T	G	V	Q	R
symbB1.v1.2.000535.t1	L	D	T	G	C	S	A	S	L	V	Q	A	N	G	S	L	L	G	D	P	K	C	N	K	G	H	G	E
symbB1.v1.2.001928.t1	C	D	T	D	G	S	S	S	L	T	N	G	Q	G	S	L	L	R	D	A	K	S	R	M	G	Y	A	T

Figure S4. Alignments of motifs within AT, ACP, KR, ER and DH domains. Active sites within the motifs are boxed with dashed lines.

AT

											*										
symbB1.v1.2.003781.t1	G	L	K	P	F	A	L	L	G	H	S	V	G	E	Y	T	A	A	V	V	A
symbB1.v1.2.007001.t1	A	T	R	F	E	A	A	A	G	L	S	L	G	E	Y	T	A	L	C	A	A
symbB1.v1.2.010404.t1	S	Q	S	W	Q	G	D	F	G	L	S	V	G	E	Y	A	A	L	T	C	A
symbB1.v1.2.011899.t1	V	E	R	C	Q	A	V	A	G	L	S	L	G	E	Y	T	A	L	A	V	A
symbB1.v1.2.012436.t1	G	L	C	L	D	V	V	L	G	H	S	N	G	E	F	M	A	A	V	A	A
symbB1.v1.2.012467.t1	V	E	R	V	Q	A	V	A	G	L	S	L	G	E	Y	T	A	L	C	A	A
symbB1.v1.2.015532.t1	A	E	D	F	R	A	V	A	G	L	S	L	G	E	Y	T	A	L	T	V	A
symbB1.v1.2.018170.t1	A	E	R	P	R	C	M	A	G	L	S	L	G	E	Y	T	A	L	C	A	A
symbB1.v1.2.022906.t1	V	D	R	A	Y	V	M	A	G	L	S	L	G	E	Y	T	A	L	C	A	A
symbB1.v1.2.025940.t1	V	D	R	P	F	C	V	A	G	L	S	L	G	E	Y	T	A	I	V	A	A
symbB1.v1.2.026318.t1	K	G	D	A	A	A	A	A	G	M	S	C	G	E	L	A	A	L	C	T	A
symbB1.v1.2.027670.t2	D	V	K	P	V	A	C	A	G	H	S	L	G	E	Y	A	A	A	V	I	G
symbB1.v1.2.030435.t1	G	V	Q	P	L	A	V	L	G	H	S	L	G	D	F	A	A	A	T	A	A
symbB1.v1.2.032358.t1	V	D	R	A	S	V	M	A	G	L	S	L	G	E	Y	T	A	L	C	A	A
symbB1.v1.2.039083.t1	G	E	V	P	S	I	V	L	G	H	S	V	G	E	I	A	A	A	Y	V	A
symbB1.v1.2.039996.t1	G	I	E	P	V	A	V	L	G	H	S	V	G	E	Y	A	A	A	V	C	C
Symbiodinium sp. C3 gil384474334	S	E	R	P	R	C	M	A	G	L	S	L	G	E	Y	T	A	L	C	A	A
Karenia brevis gblACH88363.1	G	I	E	P	G	V	F	V	G	H	S	I	G	Q	Y	V	A	G	C	L	A

ACP
(PP-binding)

									*								
symbB1.v1.2.000534.t1	P	L	T	E	M	G	L	D	S	L	A	A	A	Q	L	R	N
symbB1.v1.2.001762.t1	P	L	M	D	A	G	V	D	S	L	M	S	I	E	F	R	S
symbB1.v1.2.003781.t1	P	L	M	A	A	G	M	D	S	L	S	S	V	E	F	R	N
symbB1.v1.2.003989.t1	P	L	M	D	A	G	L	D	S	L	S	M	V	E	F	R	N
symbB1.v1.2.040551.t1	P	L	T	T	A	G	M	D	S	L	S	A	V	E	L	R	R
symbB1.v1.2.015786.t1	A	L	M	E	S	G	M	D	S	L	S	G	V	E	F	R	N
symbB1.v1.2.019479.t1	P	F	M	E	A	G	M	D	S	L	S	S	V	S	L	T	S
symbB1.v1.2.029496.t1	P	L	M	E	A	G	I	D	S	L	S	A	V	E	F	R	S
symbB1.v1.2.005063.t1	D	L	A	F	I	G	L	D	S	L	Q	A	G	V	L	V	S
symbB1.v1.2.007443.t3	T	A	L	M	D	G	I	D	S	L	G	S	T	L	V	I	N
symbB1.v1.2.008326.t1	E	F	L	C	E	N	L	D	S	L	D	L	A	R	L	Q	S
symbB1.v1.2.015788.t1	P	L	M	E	A	G	I	D	S	L	S	A	V	E	F	R	N
symbB1.v1.2.013531.t1	P	L	M	E	A	G	M	D	S	L	S	S	V	E	F	R	N
symbB1.v1.2.008780.t1	P	L	M	E	A	G	L	D	S	L	S	S	V	D	F	R	N
symbB1.v1.2.008781.t1	P	L	M	E	A	G	M	D	S	L	S	A	V	E	F	R	N
symbB1.v1.2.008782.t1	P	L	M	D	M	G	L	D	S	L	A	A	V	E	F	R	N
symbB1.v1.2.008784.t1	P	L	M	E	S	G	M	D	S	L	S	A	V	E	F	R	N
symbB1.v1.2.009497.t1	A	L	M	D	S	G	M	D	S	L	T	A	V	S	F	R	N
symbB1.v1.2.011469.t1	P	L	M	E	A	G	L	T	S	N	S	A	I	I	M	R	D
symbB1.v1.2.015740.t1	P	L	M	D	A	G	L	D	S	L	S	M	V	Q	F	R	N
symbB1.v1.2.017853.t1	P	L	M	E	S	G	L	D	S	L	S	A	V	A	F	R	N
symbB1.v1.2.038311.t1	S	L	H	Q	L	G	L	D	S	L	E	L	V	Q	L	R	R
symbB1.v1.2.035007.t1	E	F	D	E	L	G	L	D	S	L	S	G	V	E	A	S	R
symbB1.v1.2.033167.t1	A	L	M	D	I	G	L	D	S	L	A	S	V	S	F	R	D
symbB1.v1.2.036002.t1	R	I	D	I	D	G	L	E	S	L	L	M	K	N	S	A	V
symbB1.v1.2.035491.t1	A	L	M	D	A	G	L	D	S	L	A	S	V	E	F	Q	R
symbB1.v1.2.021709.t1	A	L	M	D	S	G	M	D	S	L	T	A	V	S	F	R	N
symbB1.v1.2.018367.t1	V	L	M	D	S	G	M	D	S	L	T	S	V	A	F	R	N
symbB1.v1.2.016135.t1	T	L	A	E	L	G	V	D	S	I	S	A	A	P	L	A	D
symbB1.v1.2.017506.t2	Q	D	G	L	S	G	L	E	S	L	K	V	I	I	L	V	S
symbB1.v1.2.017743.t1	D	L	N	E	S	G	L	D	S	L	A	A	A	E	L	S	T
symbB1.v1.2.018635.t1	G	F	F	A	S	G	L	D	S	F	D	M	G	V	I	Q	N
symbB1.v1.2.020351.t1	T	L	T	E	L	G	A	D	S	L	D	I	V	E	S	V	M
symbB1.v1.2.020352.t1	T	L	S	E	L	G	A	D	S	L	D	I	V	E	S	V	M
symbB1.v1.2.010769.t1	N	M	S	F	H	G	L	D	S	L	M	L	G	L	L	L	G
symbB1.v1.2.020642.t1	A	F	I	D	A	G	M	D	S	L	S	G	V	S	L	V	S
symbB1.v1.2.024742.t1	A	L	M	D	S	G	I	D	S	L	S	A	T	T	F	I	R
symbB1.v1.2.024849.t1	T	F	F	D	Y	G	M	D	S	L	E	L	I	R	V	R	R
symbB1.v1.2.025182.t1	G	F	F	Q	L	G	I	D	S	L	E	M	I	R	V	R	R
symbB1.v1.2.025887.t1	G	F	F	Q	L	G	I	D	S	L	E	M	I	R	V	K	R
symbB1.v1.2.026958.t1	P	L	M	D	M	G	L	D	S	L	S	A	M	D	L	Q	R
symbB1.v1.2.015789.t1	P	L	M	E	M	G	L	D	S	L	A	G	V	E	F	R	R
symbB1.v1.2.036576.t1	P	F	M	D	A	G	M	D	S	L	S	S	V	A	L	M	S
symbB1.v1.2.022094.t3	P	L	M	E	S	G	V	D	S	L	A	S	V	E	L	R	T
symbB1.v1.2.035340.t1	P	L	M	E	A	G	M	D	S	L	S	S	V	Q	L	V	T
symbB1.v1.2.023189.t1	P	L	M	D	A	G	L	D	S	L	A	A	V	E	F	G	R
symbB1.v1.2.030674.t3	P	L	M	D	I	G	I	N	S	M	N	A	V	V	F	R	R
symbB1.v1.2.037879.t1	P	L	M	D	M	G	L	D	S	L	S	A	V	E	F	R	R
symbB1.v1.2.026959.t1	T	L	L	R	S	G	M	D	S	L	S	G	V	E	F	R	R
symbB1.v1.2.040289.t1	F	V	H	D	L	G	I	D	S	A	G	M	A	G	L	L	G
symbB1.v1.2.029823.t1	T	F	E	D	A	G	L	D	S	L	S	L	I	S	L	A	R
symbB1.v1.2.034871.t1	V	F	V	E	V	G	V	D	S	I	T	A	V	D	L	V	R

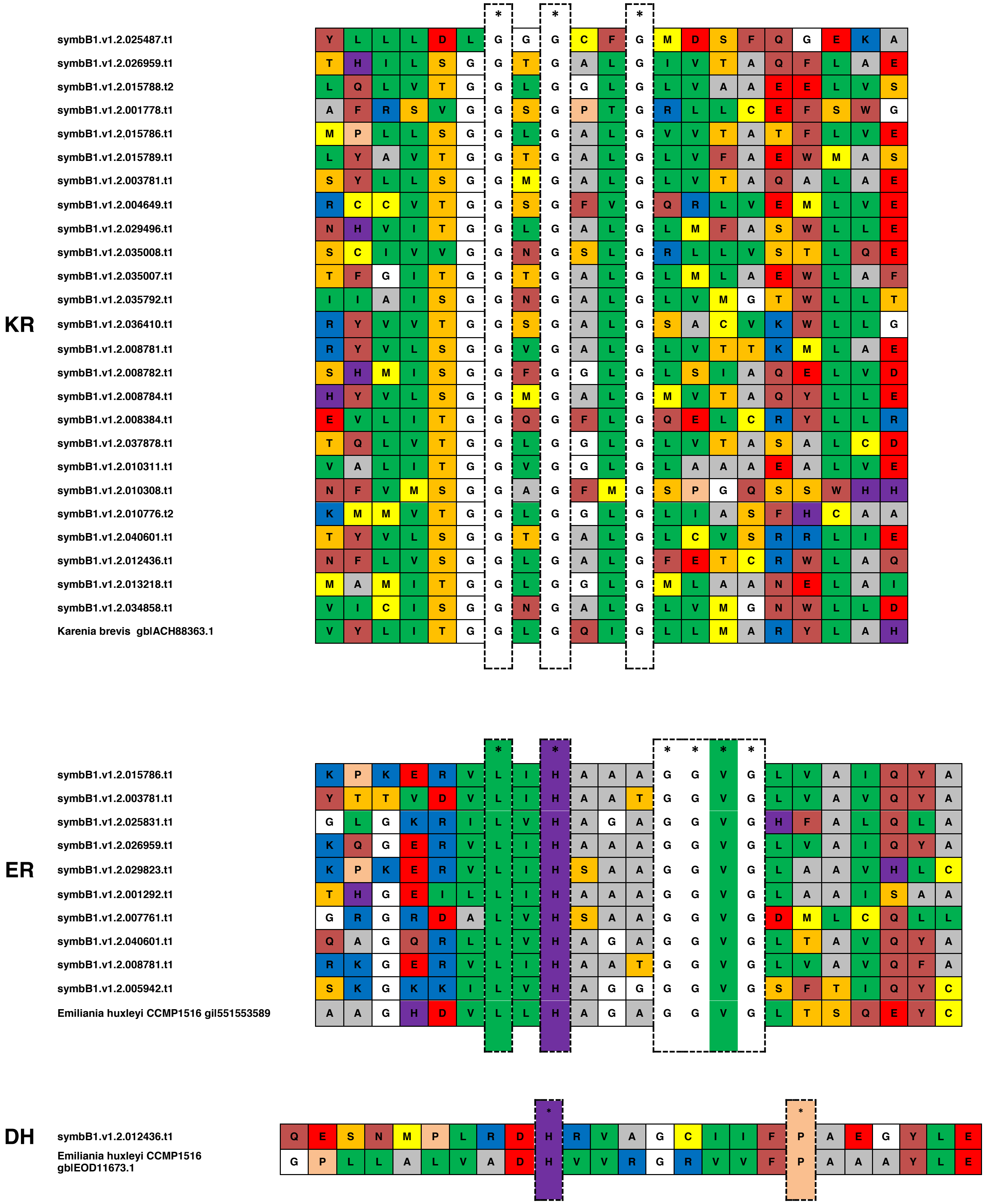
KR
symbB1.v1.2.025487.t1 Y L L L D L G G G C F G M D S F Q G E K A
symbB1.v1.2.026959.t1 T H I L S G G T G A L G I V T A Q F L A E
symbB1.v1.2.015788.t2 L Q L V T G G L G G L G L V A A E E L V S
symbB1.v1.2.001778.t1 A F R S V G G S G P T G R L L C E F S W G
symbB1.v1.2.015786.t1 M P L L S G G L G A L G V V T A T F L V E
symbB1.v1.2.015789.t1 L Y A V T G G T G A L G L V F A E W M A S
symbB1.v1.2.003781.t1 S Y L L S G G M G A L G L V T A Q A L A E
symbB1.v1.2.004649.t1 R C C V T G G S G F V G Q R L V E M L V E
symbB1.v1.2.029496.t1 N H V I T G G L G A L G L M F A S W L L E
symbB1.v1.2.035008.t1 S C I V V G G N G S L G R L L V S T L Q E
symbB1.v1.2.035007.t1 T F G I T G G T G A L G L M L A E W L A F
symbB1.v1.2.035792.t1 I I A I S G G N G A L G L V M G T W L L T
symbB1.v1.2.036410.t1 R Y V V T G G S G A L G S A C V K W L L G
symbB1.v1.2.008781.t1 R Y V L S G G V G A L G L V T T K M L A E
symbB1.v1.2.008782.t1 S H M I S G G F G G L G L S I A Q E L V D
symbB1.v1.2.008784.t1 H Y V L S G G M G A L G M V T A Q Y L L E
symbB1.v1.2.008384.t1 E V L I T G G Q G F L G Q E L C R Y L L R
symbB1.v1.2.037878.t1 T Q L V T G G L G G L G L V T A S A L C D
symbB1.v1.2.010311.t1 V A L I T G G V G G L G L A A A E A L V E
symbB1.v1.2.010308.t1 N F V M S G G A G F M G S P G Q S S W H H
symbB1.v1.2.010776.t2 K M M V T G G L G G L G L I A S F H C A A
symbB1.v1.2.040601.t1 T Y V L S G G T G A L G L C V S R R L I E
symbB1.v1.2.012436.t1 N F L V S G G L G A L G F E T C R W L A Q
symbB1.v1.2.013218.t1 M A M I T G G L G G L G M L A A N E L A I
symbB1.v1.2.034858.t1 V I C I S G G N G A L G L V M G N W L L D
Karenia brevis gb|ACH88363.1 V Y L I T G G L G Q I G L L M A R Y L A H
ER
symbB1.v1.2.015786.t1 K P K E R V L I H A A A G G V G L V A I Q Y A
symbB1.v1.2.003781.t1 Y T T V D V L I H A A T G G V G L V A V Q Y A
symbB1.v1.2.025831.t1 G L G K R I L V H A G A G G V G H F A L Q L A
symbB1.v1.2.026959.t1 K Q G E R V L I H A A A G G V G L V A I Q Y A
symbB1.v1.2.029823.t1 K P K E R V L I H S A A G G V G L A A V H L C
symbB1.v1.2.001292.t1 T H G E I L L I H A A A G G V G L A A I S A A
symbB1.v1.2.007761.t1 G R G R D A L V H S A A G G V G D M L C Q L L
symbB1.v1.2.040601.t1 Q A G Q R L L V H A G A G G V G L T A V Q Y A
symbB1.v1.2.008781.t1 R K G E R V L I H A A T G G V G L V A V Q F A
symbB1.v1.2.005942.t1 S K G K K I L V H A G G G G V G S F T I Q Y C
Emiliania huxleyi CCMP1516 gi|551553589 A A G H D V L L H A G A G G V G L T S Q E Y C
DH
symbB1.v1.2.012436.t1 Q E S N M P L R D H R V A G C I I F P A E G Y L E
Emiliania huxleyi CCMP1516 gb|EOD11673.1 G P L L A L V A D H V V R G R V V F P A A A Y L E

Figure S5. (A) Multiple alignment of the truncated, conserved, N-termini of dinoflagellate KSs (B) Additional Symbiodinium sequences with the divergent signatures. Lower panel: Maximum likelihood tree of the N-termini computed with 1000 bootstrap replicates. Bootstrap values $\geq$ 50% are marked at appropriate nodes.

A

**A.spinosum AS3D902**	L	G	R	L	A	Q	E	V	E	E	G	Y	L	G	K	N	G	R	A	K	V	M	W	L	D
**A. spinosum AS3D901**	–	–	–	–	–	–	–	–	–	–	–	–	–	–	–	–	–	–	–	–	–	M	W	L	D
**A.ostenfeldii Ac0019**	F	G	R	L	C	M	E	V	E	E	G	Y	L	G	K	N	G	R	A	K	V	M	W	V	D
**A. spinosum AS3D906**	L	Q	R	F	P	E	E	V	E	E	G	Y	L	G	Q	G	G	R	G	K	V	A	W	M	D
**H. triquetra 5908**	L	F	R	L	P	E	E	V	E	E	G	Y	L	G	F	G	A	K	G	K	V	A	W	L	D
**K. brevis KB5361**	F	V	R	L	P	S	E	L	E	P	G	Y	L	G	K	D	C	T	A	K	T	M	N	L	D
**K. brevis KB2006**	F	G	R	L	A	V	E	F	E	P	G	Y	L	G	R	G	A	G	A	K	V	L	H	L	D
**A. ostenfeldii Ac0038**	F	S	R	L	A	V	E	F	E	R	G	Y	L	G	K	E	G	S	A	K	T	L	L	L	D
**H. triquetra 6310**	F	S	R	L	A	V	E	F	E	S	G	Y	L	G	K	E	S	T	A	K	T	L	L	L	D
**symbB1.v1.2.019160.t1**	F	A	R	L	P	T	E	F	E	L	G	Y	L	G	R	G	S	R	T	D	R	V	A	M	L
**symbB1.v1.2.022565.t1**	S	S	A	L	R	R	E	F	L	P	D	Y	L	G	K	D	S	H	S	K	V	G	I	L	D
**symbB1.v1.2.001307.t1**	W	G	V	F	P	S	E	T	E	E	E	Y	L	G	A	S	Q	N	R	L	R	M	F	D	I
**symbB1.v1.2.028834.t1**	F	F	R	M	K	Q	E	V	E	T	G	Y	M	G	F	D	S	N	N	K	I	A	W	L	Q
**A. ostenfeldii 10-X J14**	F	E	K	M	K	A	E	F	E	V	G	Y	L	G	N	E	N	I	T	R	C	A	V	I	E
**A. spinosum AS3D903**	Y	E	G	L	R	A	E	M	E	E	A	F	L	G	K	E	N	N	S	R	V	G	L	I	E

B

**symbB1.v1.2.008781.t1**	A	K	S	V	V	L	L	S	R	R	G	V	V	G	D	D	L	K	S	M	W	D	K	L	Q
**symbB1.v1.2.015788.t2**	V	L	F	S	S	V	S	A	T	I	G	L	A	G	G	A	S	Y	A	A	A	N	C	Y	L
**symbB1.v1.2.036410.t1**	S	S	H	L	T	L	F	L	V	G	G	A	M	G	R	V	E	T	S	F	G	D	F	A	Q
**symbB1.v1.2.027671.t1**	L	Y	V	G	G	V	V	L	S	Y	G	Y	Y	G	N	S	D	I	T	A	A	K	F	S	P
**symbB1.v1.2.000535.t1**	F	A	N	V	G	N	H	T	Q	A	G	Y	V	V	A	F	I	D	R	E	M	N	D	G	K
**symbB1.v1.2.036002.t1**	L	V	I	T	G	W	N	V	A	D	G	Y	L	P	P	A	K	S	T	S	F	K	R	N	D
**symbB1.v1.2.013880.t1**	L	T	E	D	Y	L	V	Q	G	G	G	C	E	G	D	V	E	L	T	E	A	D	C	S	E
**symbB1.v1.2.001928.t1**	L	K	Q	P	P	A	M	V	H	D	G	L	L	G	E	E	G	S	S	L	I	L	D	L	F
**symbB1.v1.2.015913.t1**	W	Q	Q	A	N	T	V	I	Q	E	G	L	L	T	E	G	S	A	F	I	A	E	L	E	S
**symbB1.v1.2.030435.t1**	A	L	A	S	S	G	H	D	Q	A	T	L	L	G	S	P	M	S	I	F	V	A	L	S	N

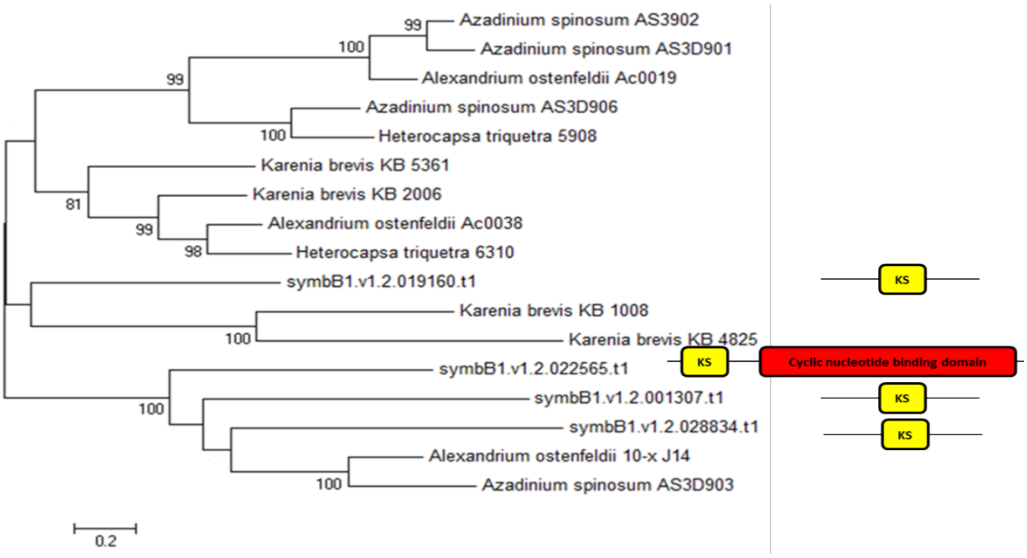

Supplement: Additional file 2: — Figure S3. (A) Alignment of KS domains with those of other dinoflagellates and animal PKSs and FASs. Asterisks indicate conserved amino acids required for catalytic activity. (B) Additional Symbiodinium and K. brevis sequences that are consistent with previous reports. Figure S4. Alignments of motifs within AT, ACP, KR, ER, and DH domains. Active sites within the motifs are boxed with dashed lines. Figure S5. (A) Multiple alignment of the truncated, conserved, N-termini of dinoflagellate KSs (B) Additional Symbiodinium sequences with the divergent signatures. Lower panel: Maximum likelihood tree of the N-termini computed with 1000 bootstrap replicates. Bootstrap values ≥ 50 % are marked at appropriate nodes. (PDF 2705 kb) [file 12864_2015_2195_MOESM2_ESM.pdf]
