# Supplementary material for: Multifunctional polyketide synthase genes identified by genomic survey of the symbiotic dinoflagellate, Symbiodinium minutum
Source: BMC Genomics. 2015 Nov 14;16:941. doi: 10.1186/s12864-015-2195-8 (PMC4647583; doi:10.1186/s12864-015-2195-8)

Figure S6. (A) NanoLC-MS (positive ion) profile of the methanol extract of *Symbiodinium minutum*. Top Chromatogram, Center Top: Extract ion (*m/z* 1072.60, 10.6 min), Center bottom: (*m/z* 1050.57, 10.6 min), Bottom: MS spectrum. (B) MS spectrum (positive ion) of the methanol extract (expanded).

A

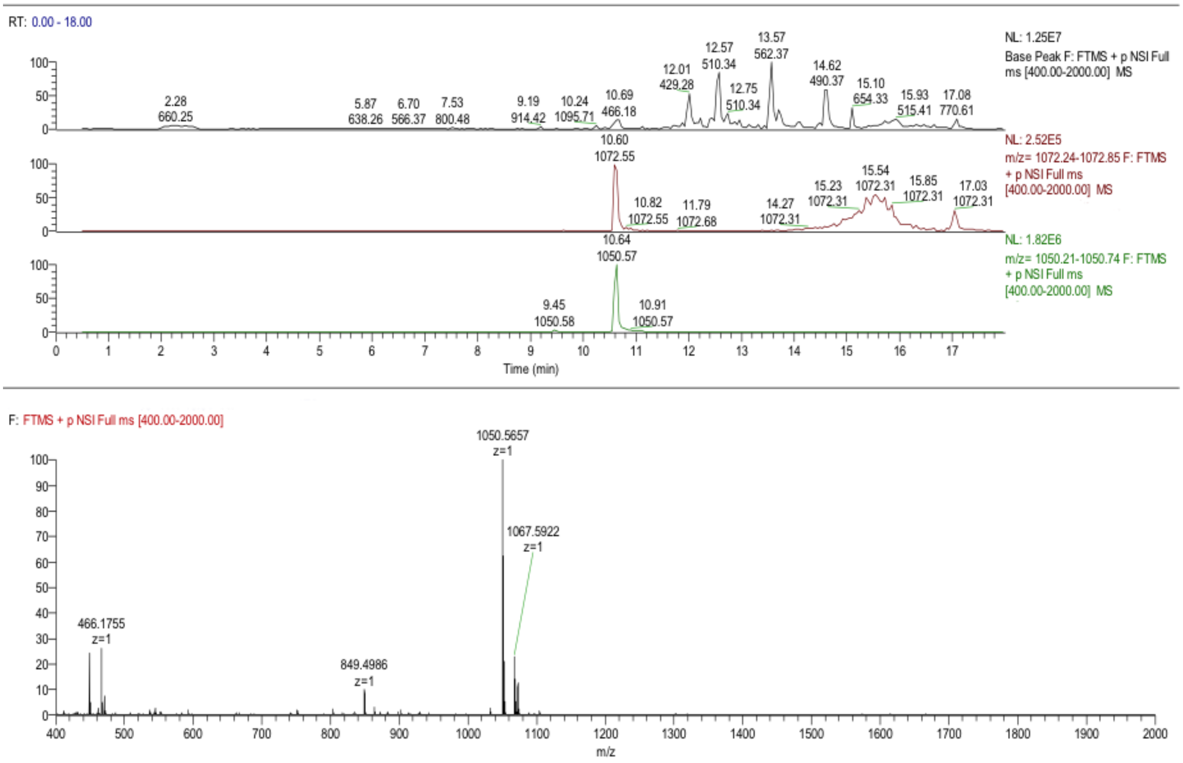

B

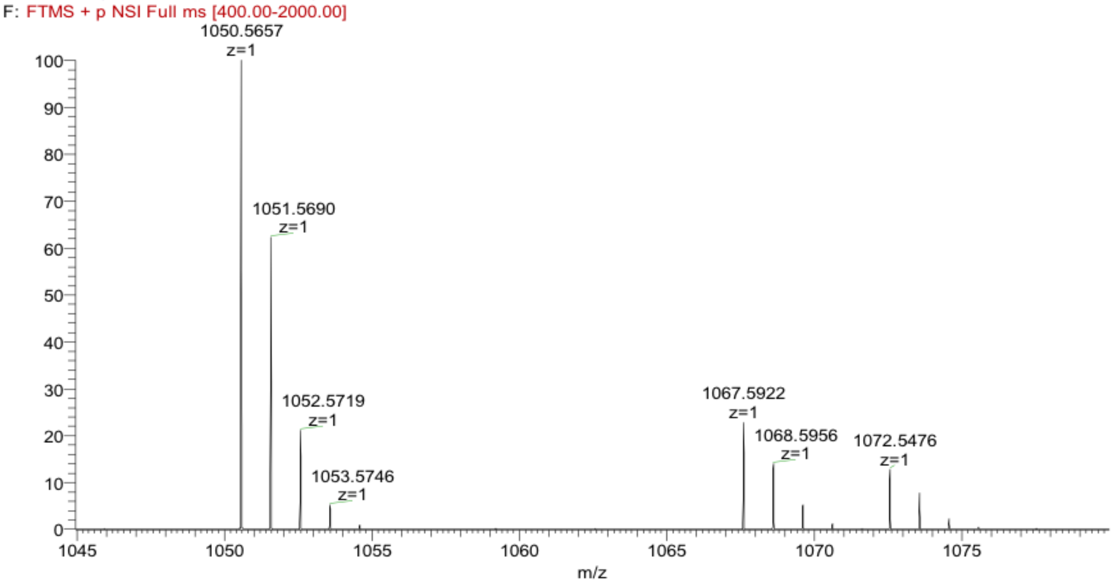

Supplement: Additional file 3: — Figure S6. (A) NanoLC-MS (positive ion) profile of the methanol extract of Symbiodinium minutum. Top Chromatogram, Center Top: Extract ion (m/z 1072.60, 10.6 min), Center bottom: (m/z 1050.57, 10.6 min), Bottom: MS spectrum. (B) MS spectrum (positive ion) of the methanol extract (expanded). (PDF 145 kb) [file 12864_2015_2195_MOESM3_ESM.pdf]
